# Evaluation of Low-Cost Mitigation Measures Implemented to Improve Air Quality in Nursery and Primary Schools

**DOI:** 10.3390/ijerph14060585

**Published:** 2017-05-31

**Authors:** Juliana P. Sá, Pedro T. B. S. Branco, Maria C. M. Alvim-Ferraz, Fernando G. Martins, Sofia I. V. Sousa

**Affiliations:** LEPABE—Laboratory for Process Engineering, Environment, Biotechnology and Energy, Faculty of Engineering, University of Porto, Rua Dr. Roberto Frias, 4200-465 Porto, Portugal; julianasa@fe.up.pt (J.P.S.); p.branco@fe.up.pt (P.T.B.S.B.); aferraz@fe.up.pt (M.C.M.A.-F.); fgm@fe.up.pt (F.G.M.)

**Keywords:** indoor air, mitigation measures evaluation, nursery schools, primary school

## Abstract

Indoor air pollution mitigation measures are highly important due to the associated health impacts, especially on children, a risk group that spends significant time indoors. Thus, the main goal of the work here reported was the evaluation of mitigation measures implemented in nursery and primary schools to improve air quality. Continuous measurements of CO_2_, CO, NO_2_, O_3_, CH_2_O, total volatile organic compounds (VOC), PM_1_, PM_2.5_, PM_10_, Total Suspended Particles (TSP) and radon, as well as temperature and relative humidity were performed in two campaigns, before and after the implementation of low-cost mitigation measures. Evaluation of those mitigation measures was performed through the comparison of the concentrations measured in both campaigns. Exceedances to the values set by the national legislation and World Health Organization (WHO) were found for PM_2.5_, PM_10_, CO_2_ and CH_2_O during both indoor air quality campaigns. Temperature and relative humidity values were also above the ranges recommended by American Society of Heating, Refrigerating, and Air-Conditioning Engineers (ASHRAE). In general, pollutant concentrations measured after the implementation of low-cost mitigation measures were significantly lower, mainly for CO_2_. However, mitigation measures were not always sufficient to decrease the pollutants’ concentrations till values considered safe to protect human health.

## 1. Introduction

Children usually spend 80–90% of their time indoors where they are exposed to higher levels of air pollution than those from outdoor air [1,2,3]. They are considered a risk group because they are more vulnerable to air pollution than adults [4,5,6]. Among indoor environments, nursery and primary schools need a special attention because children spend more time there than in any other indoor environment besides home. Moreover, several studies have recognized a relationship between indoor air quality (IAQ) and adverse health effects on children, namely respiratory illness and poor cognitive performance [5,7,8,9,10,11,12,13].

It is known that a poor IAQ depends on several factors, of which it can be highlighted the use of high emitting materials for building construction and furnishing, minimal landscaping with poor drainage, heating, ventilation and air conditioning units, the lack of preventative maintenance, crowded conditions and cleaning products that release chemicals into the air [2,14,15,16,17].

After observing high concentrations of indoor air pollutants, mainly particulate matter (PM), carbon dioxide (CO_2_) and volatile organic compounds (VOC), several studies concluded that there is a pressing need to implement strategies to improve IAQ, through the implementation of measures to mitigate indoor air pollution (IAP): (i) the change of some behavioural habits, promoting efficient ventilation [8,12,18,19,20,21,22,23,24,25,26,27,28,29,30,31]; (ii) the improvement of cleaning actions [19,21,22,23,27,29,31,32,33]; (iii) the replacement of carpets and carpeted floors by smooth panels [27,33]; (iv) the adequacy of occupational density and promotion of more class breaks and outdoor activities [12,27,32]; and (v) structural measures such as the installation of air purifiers [33], the replacement of building materials, furniture and windows [25,34] and the replacement of the heating system [26].

Some government organizations and programs developed measures, guidelines and/or regulations aiming to improve IAQ in school settings, such as the International Society of the Indoor Air Quality and Climate (ISIAQ) [35] and the European Federation of Allergy and Asthma Associations (EFA) [36]. Also the World Health Organization (WHO) has published guidelines, reference levels and recommendations for IAQ, which although generic can be applied to the school context [37]. In addition, SINPHONIE project developed guidelines and general recommendations for IAQ improvement in the most diverse microenvironments (ME) of a school, such as classrooms, gymnasiums, laboratories, lunch and dressing rooms (ranging from requirements for structures and building materials to guidelines for education and awareness of building occupants) [38]. On the other hand, the United States Environmental Protection Agency (USEPA) has also been working on the implementation of IAQ mitigation measures by distributing an action kit called “Tools for Schools Action Kit” to public schools, teachers and health professionals, as well as to students and their parents/guardians [39].

It is clear the relevance of developing and implementing guidelines and measures to mitigate IAP in nursery and primary schools. However, the evaluation of the impacts of implementing mitigation measures is yet very scarce. As far as the authors’ knowledge goes, only one study, conducted in the framework of the Forced-ventilation Related Environmental School Health (FRESH) project quantified a significant decrease in mean CO_2_, endotoxin, β(1,3)-glucan and PM_10_ levels after the implementation of mechanical ventilation systems in 18 classrooms of 17 primary schools in the north of The Netherlands [40,41] (it should be remarked that PM_2.5_ and NO_2_ concentrations didn’t decrease). To fulfil this gap, the main goal of the work here reported was evaluating IAP mitigation measures implemented in nursery and primary schools. Thus, this study is the first that focuses on the hierarchy application and quantification of IAP mitigation measures, centred on low-cost and easy to apply measures. In addition, unlike the study above referred this study also focuses on nursery schools, the first social environment of children, because early exposure to air pollution might have significant impact on children’s health [19,42].

## 2. Materials and Methods

### 2.1. Sites Description and IAQ Characterization

This study was carried out in three different buildings (1, 2 and 3) located in urban and suburban areas in Porto district, Portugal. The selection of study locations was based in the INAIRCHILD project [19,20,21,22,23,43,44,45,46,47,48]. A total of sixteen ME from two nursery schools for infants (CR1 and CR3) and other two for pre-schoolers (JI1 and JI2), as well as two primary schools (PRIM1 and PRIM2) were studied. Table 1 shows a general description of each studied ME, namely its use, class/grade, building floor, area, occupancy and period of occupation. Relevant information on operating mode and activities, characteristics of the building and of the ME and potential sources of pollution were gathered by a previous inspection to the different schools (throughout observations and interviews with the staff). 

In nursery schools for infants (children aged under 3 years old) activities were more restrained due to the low mobility of this age group, and the daily pattern included a sleeping time after lunch (nap). The youngest children (<1 year-old) spent all the occupation period inside the classroom (CR1_A) including sleeping and eating, while children from CR3 occupied different ME (classrooms, lunch rooms, sleeping room). On the other hand, in nursery schools for pre-schoolers (aged 3–5 years old) children were usually more active and used a great diversity of materials (e.g., paints, glues). In primary schools (children from 1st to 4th grade, aged between 6 and 10 years old) occupants usually stood seated at the desks during classes. All the schools had an outdoor playground.

Electric heaters were constantly used in buildings 1 and 3 and natural ventilation was predominant in all ME. Although building 2 had both electric heating and mechanical ventilation systems, those were not used during the study period. General cleaning activities were usually made at the end of the occupation period in all the studied ME. In some cases, cleaning was also made during the lunch time or before nap.

To characterize IAQ, concentrations of gaseous compounds, namely CO_2_ (carbon dioxide), carbon monoxide (CO), nitrogen dioxide (NO_2_), ozone (O_3_), formaldehyde (CH_2_O) and total volatile organic compounds (TVOC), as well as levels of comfort parameters, temperature (T) and relative humidity (RH) were sampled using an Haz-Scanner IEMS (SKC Inc., Eighty Four, PA, USA). Indoor concentrations of different PM fractions (PM_1_, PM_2.5_, PM_10_ and TSP) were also sampled using a TSI DustTrak™ DRX 8534 Aerosol Monitor (TSI, Shoreview, MN, USA). Moreover, radon concentrations were sampled using a Radim 5B monitor (SMM, Prague, Czech Republic). Equipment were submitted to a standard zero calibration (available in the equipment) and data were validated prior to each measurement in the different rooms. The equipment was placed inside the ME, exactly in the same position (in both campaigns), avoiding to disturb the normal functioning of school activities, as close to the centre as possible, far from windows, doors and corners, approximately at the same height of the breathing zone of children. The sampling methodology, as well as the main characteristics of each equipment, including the associated errors were previously described in detail [19,20,21,22,23,43]. IAQ sampling was performed continuously between 17 February and 1 June 2016, in two campaigns, and consecutively at least during a complete day in each ME, and not exceeding two consecutive weekdays, since studies for longer periods had already been carried out applying the same methodology [19,20,21,22,23]. Although the measurement period took place at different seasons, both campaigns were carried out with the same physical characteristics of the ME, in the same school year and consequently with the same occupation rate, same schedule and activity patterns. In some cases, measurements were performed both on weekdays and weekends for occupation/non-occupation comparison. All samplings were logged each minute, with the exception of PM (15-min) and Rn (1 h).

### 2.2. Evaluation of Mitigation Measures

Evaluation of mitigation measures was performed through the comparison of the concentrations measured in two campaigns: before and after the implementation of low-cost mitigation measures. Given the difficulty in setting a control in this type of fieldwork (conditions may be very variable) the first campaign, before the implementation of mitigation measures was considered as the reference (control). Measured concentrations were compared with standard values (WHO and/or Portuguese legislation). 

In order to improve IAQ, several IAP mitigation measures were identified, based on the literature [8,12,18,19,20,21,22,23,24,25,26,27,28,29,30,31,38,39] and grouped hierarchically in 5 different types, from the less to the most expensive and complex: Type I—raising awareness; Type II—behavioural changes; Type III—changes in products/materials and places of activities; Type IV—technical and technological changes; Type V—structural changes. Detailed description of those measures can be consulted in Table S1 (Supplementary Materials). It should be noted that this methodology is an initial approach to the quantification of the application of IAP mitigation measures.

Results from the first campaign, allowed identifying IAP mitigation measures to be specifically implemented in each ME, and potentially extended to other ME in the studied buildings. Schools’ coordinators were actively involved in the implementation of mitigation measures. Existing good practices were encouraged to be maintained. Mitigation measures were selected and delivered to the schools’ coordinators that coordinated their implementation. The staff responsible by the implementation of mitigation measures in each studied ME received a daily log to fill in, informing about the application or not of the selected IAP mitigation measures. 

### 2.3. Comparison with Standard Values

Comparisons with standard values allowed evaluating exceedances and/or non-compliances. For Portuguese legislation [49], running mean values were calculated for the period of occupation, non-occupation and weekend, and the maximum value (for each period) was compared with the limit for health protection: (i) 2250 mg/m^3^ for CO_2_; (ii) 600 µg/m^3^ for TVOC; (iii) 100 µg/m^3^ for CH_2_O; (iv) 10 µg/m^3^ for CO; (v) 25 µg/m^3^ and 50 µg/m^3^ for PM_2.5_ and PM_10_, respectively; and (vi) 400 Bq/m^3^ for Rn. As there were no mechanical ventilation systems being used, a margin of tolerance was added for CO_2_ (30%), and for TVOC, PM_2.5_, and PM_10_ (100%), as required by legislation. In turn, 30-minutes CH_2_O, hourly CO and NO_2_ and daily PM_2.5_ and PM_10_ mean values were determined for comparison with the respective guidelines recommended by WHO [37,50]: (i) 100 µg/m^3^ for CH_2_O; (ii) 35 mg/m^3^ for CO; (iii) 200 mg/m^3^ for NO_2_; (iv) 25 µg/m^3^ and 50 µg/m^3^ for PM_2.5_ and PM_10_, respectively; and (v) 100 Bq/m^3^ for Rn. T and RH hourly means were also compared with American Society of Heating, Refrigerating, and Air-Conditioning Engineers (ASHRAE) standard reference ranges (T: 20.0–23.6 °C and 22.8–26.1 °C, for winter and summer seasons, respectively; RH: 30–65%) [51]. 

### 2.4. Statistical Analysis

Hourly mean and median values were calculated for all the studied pollutants and comfort parameters. The non-parametric Wilcoxon Signed Rank Test was used to analyse if the differences along the day were significant [52]; the non-parametric Wilcoxon Rank Sum Test (also called Mann–Whitney U test) was used to analyse the significance of the differences between hourly mean in different days for each ME, between measurement campaigns, between weekdays and weekends, as well as between different ME and schools [53]. On the other hand, associations between different pollutants were performed applying Spearman correlation [54]. In all cases, a significance level (α) of 0.05 was considered. Descriptive statistics for the parameters were calculated using MS Excel^®^ (Microsoft Corporation, Redmond, DC, USA), and all other statistical analysis were determined using R software, version 3.3.0 [55].

## 3. Results and Discussion

### 3.1. IAQ Characterization

As there were no statistically significant differences (*p* > 0.05) on IAP between consecutive weekdays, and as the daily pattern during the different sampling weekdays in each ME appeared to be similar, mean daily profiles were performed to represent mean IAQ scenarios for both weekdays and weekends for all pollutants, similarly to what was previously reported by Branco, et al. [20], Branco, et al. [21] and Nunes, et al. [23]. This was done for both campaigns (before and after IAP mitigation measure implementation). Daily profiles considering the 24-h period along weekdays and weekend allowed to understand differences between occupation and non-occupation periods, which contributed to sources identification for all the evaluated parameters and air pollutants. 

#### 3.1.1. Comfort Parameters: Temperature and Relative Humidity

**Figure 1:**
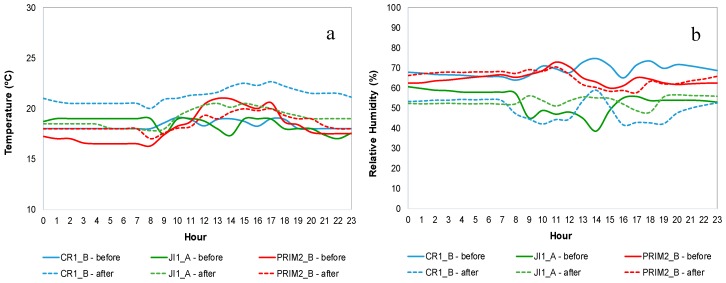
Comfort parameters mean daily profiles for CR1_B, JI1_A and PRIM2_B: (**a**) Temperature; (**b**) Relative humidity.

Figure 1 shows T and RH mean daily profiles for CR1_B, JI1_A and PRIM2_B (as example), during both IAQ campaigns, before mitigation measures (continuous line) and after mitigation measures (dashed line). Figures S1 and S2 (Supplementary Materials) show T and RH mean daily profile, respectively, for both IAQ campaigns in all buildings.

On the weekend and non-occupation periods, both T and RH showed no significant variations along the day, and usually values were lower than on weekdays. The daily patterns of both comfort parameters during weekdays were characterized by a slight increase usually observed at the beginning of the occupation period and a decrease after the end. Similar trends were reported by Dorizas, et al. [56] and Branco, et al. [20]. The observed increase in T appeared to be related with the use of electric heating (building 1 and 3—first IAQ campaign) and with occupation. On the other hand, the pattern observed in RH seemed to be related with the influence from outdoor air and with occupation. In PRIM2_A and PRIM2_C, it occurred a significant increase of RH at the end of the occupation period, in the second campaign (*p* < 0.05). This fact seemed to be related not only with the influence of the outdoor air (rainy days), but also with deep cleaning using watery and aqueous products. Regarding lunch rooms (RF1 and RF3), RH peaks were observed during the cooking process.

#### 3.1.2. Particulate Matter

**Figure 2:**
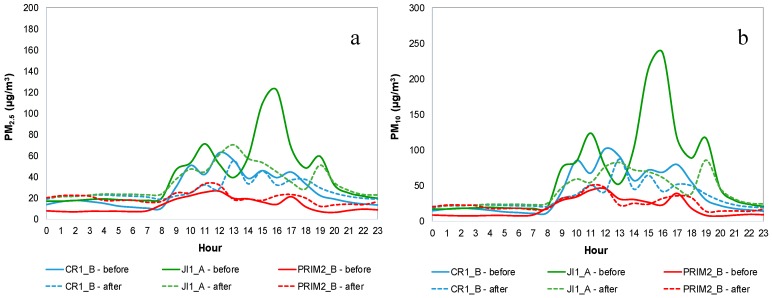
Mean daily profile for CR1_B, JI1_A and PRIM2_B: (**a**) PM_2.5_; (**b**) PM_10_.

Figure 2 show PM_2.5_ and PM_10_ mean daily profile for CR1_B, JI1_A and PRIM2_B (as example), during both IAQ campaigns, before mitigation measures (continuous line) and after mitigation measures (dashed line). Figures S3–S6 (Supplementary Materials) show PM_1_, PM_2.5_, PM_10_ and TSP mean daily profile, respectively, for both IAQ campaigns in all buildings. 

It was possible to identify, for all size fractions and for both IAQ campaigns, a similar profile pattern for PM concentrations at the classrooms of all the studied school buildings, characterized by an increase at the beginning of the occupation period, followed by a slight decrease during lunch time, period when children moved to the common lunch room; after lunch time, when children returned to the classrooms, a new increase was observed, which was maintained until the end of the occupation period. In most of the studied ME, it was also possible to identify a slight increase in the concentration of all PM fractions at the end of occupation period, which could have been related to the cleaning activities, decreasing during night and dawn. The lowest concentrations were registered during non-occupation periods, when there were no wide variations neither peaks of concentration. On the other hand, in lunch rooms (RF1 and RF3), PM concentrations increased during lunch time due to the great number of occupants (children) present in the same room at the same time, and during and immediately after the snack time due to the cleaning activities (more evident in RF1). Although these were the ME more occupied, they were also those with the largest area, resulting in lower concentrations when comparing to other ME. In sleeping rooms (CR3_D1 and CR3_D2) an increase in PM concentrations immediately before and after the nap was observed, possibly due to re-suspension phenomena caused by the preparation of the room and children. 

In building 1, during the first campaign, the highest PM concentrations from all size fractions were observed in the classroom with the highest occupation (JI1_A), probably due to an elevated re-suspension caused by occupants’ activities. Canha, et al. [26], Branco, et al. [19], Mainka and Zajusz-Zubek [27], Fuoco, et al. [57] and Nunes, et al. [22] have also identified the high number of occupants as a contributing factor to increase the re-suspension of particles in indoor air. Furthermore, Quirós-Alcalá, et al. [7] identified indoor ventilation and building characteristics as the main causes for the PM concentrations observed (max = 128 and 207 µg/m^3^ for PM_2.5_ and PM_10_, respectively). In the second campaign results from JI1_A were not considered, due to an atypical situation: the presence of about 60 people in the room during the morning—children, their parents and school’s staff—and the absence of people during the afternoon).

Regarding building 2, in the first campaign, a higher peak was identified in the late afternoon in PRIM2_C, mainly for coarser fractions (TSP and PM_10_), which it is thought to be related with deep cleaning activities, associated with the presence of a chalkboard. Dorizas, et al. [56] concluded that the use of chalkboards in classrooms led to increased PM levels in indoor air and Canha, et al. [26] identified the presence of chalkboard as one of the main PM sources in classrooms.

In building 3, during the first campaign, the highest PM concentrations were found in CR3_A. Those concentrations remained high in the second campaign, which was somewhat expected since the period of occupation started earlier than in the other ME, and all the daily activities were carried out inside the same classroom (including child’s reception before classes, playground, sleeping/nap, activities after classes). This activity pattern led to earlier and continuous re-suspension phenomena, which promoted higher PM concentrations. Semi-open windows at night, plus gardening activities in the building’s backyard, may have caused the increased PM concentrations in CR3_C observed in the second campaign.

In general, classrooms for pre-schoolers (JI1_A and JI2_A) were those with the highest levels of PM concentrations in all fractions, mainly due to the type of activities and the increased mobility of children, which contributed substantially to the suspension and/or re-suspension of particles. Mainka and Zajusz-Zubek [27] and Branco, et al. [19] also concluded that higher PM concentrations were usually found in classrooms for pre-schoolers, for all fractions, while Nunes, et al. [22] only reported this in coarser fractions.

#### 3.1.3. CO_2_

**Figure 3:**
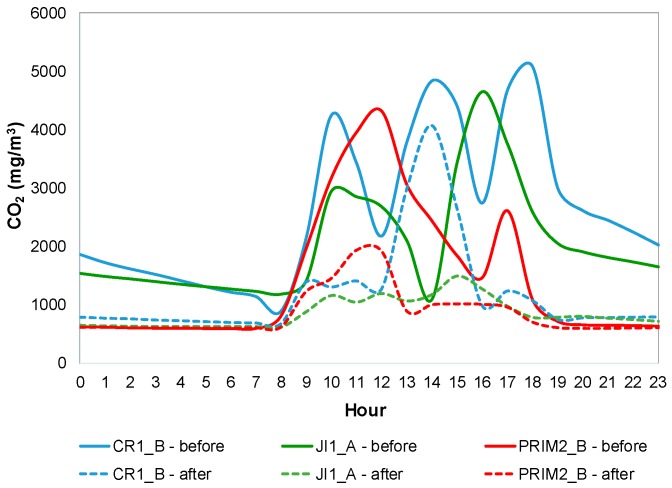
CO_2_ mean daily profile for CR1_B, JI1_A and PRIM2_B.

Figure 3 shows CO_2_ mean daily profile for CR1_B, JI1_A and PRIM2_B (as example), during both IAQ campaigns, before mitigation measures (continuous line) and after mitigation measures (dashed line). Figure S7 (Supplementary Materials) shows CO_2_ mean daily profile for both IAQ campaigns in all buildings.

In most of the studied ME, two peaks of CO_2_ concentrations were observed: (i) in the morning, with an increase at the beginning of the occupation period, followed by a decrease immediately before lunch time; and (ii) in the afternoon, with an increase after lunch, followed by a decrease at the end of the occupation period. In lunch rooms, the highest concentrations were registered during lunch and afternoon snack (when they were occupied) which matched the decreases of concentrations in classrooms. CR3_B and CR3_C presented only a peak during the morning, and CR3_D1 and CR3_D2 presented only one peak during the afternoon, both corresponding to their occupation periods (Table 1). A statistically significant difference (*p* < 0.05) was observed between weekdays and weekends. On weekends and non-occupation periods concentrations were usually below 2000 mg/m^3^ in the first campaign and below 1000 mg/m^3^ in the second (Figure S7).

ME from building 2 were those with the highest CO_2_ concentrations (567–5349 mg/m^3^), which seemed to be related to the lack of ventilation, especially during the first campaign, since natural ventilation through windows and/or doors opening was practically not performed. This not only led to a continuous accumulation of CO_2_ inside the classrooms, but also prevented its dispersion. These CO_2_ concentrations were also higher than those reported by other previous studies [12,24,32,34]. However, Dorizas, et al. [56], in a study conducted in nine primary schools in Athens reported maximum CO_2_ values of 9368 mg/m^3^.

CR3_D1 was the ME with the highest CO_2_ concentrations in building 3 (max = 3589 mg/m^3^), which may be related with the higher occupation, when compared with the other studied ME in the same building, and to its design, since natural ventilation was only made through two doors to the inner corridor. In CR1_A CO_2_ pattern seemed different, with weaker variations along the day, as children spent all day inside the same room (all activities occurred in the same room, including the lunch and sleeping/nap). The highest CO_2_ concentrations in the ME for infants were found during the sleeping/nap time, both in classrooms (CR1_A, CR1_B, CR3_A) and in sleeping rooms (CR3_D1 and CR3_D2), due to the lowest ventilation during that time (windows and doors were closed to maintain the silence). 

Globally, in the first campaign, ME occupied by younger children (infants and pre-schoolers) had higher concentrations than primary schools’ ME, which seemed to be related with longer occupation periods (except in CR3_B and CR3_C) and less ventilation (due to higher susceptibility of younger children to get cold).

Although lack of ventilation seemed to be the factor that most influenced CO_2_ concentrations in all ME, occupation density (number of children per area of each ME) was also concerning and a determining factor. Similarly, Branco, et al. [20] studied comfort parameters and CO_2_ concentration in 4 nurseries of Porto district and also identified poor ventilation and high number of occupants (children) as the main causes for high CO_2_ concentrations. Turanjanin, et al. [24], Lazović, et al. [29] and Nunes, et al. [23] concluded the same.

#### 3.1.4. TVOC and CH_2_O

**Figure 4:**
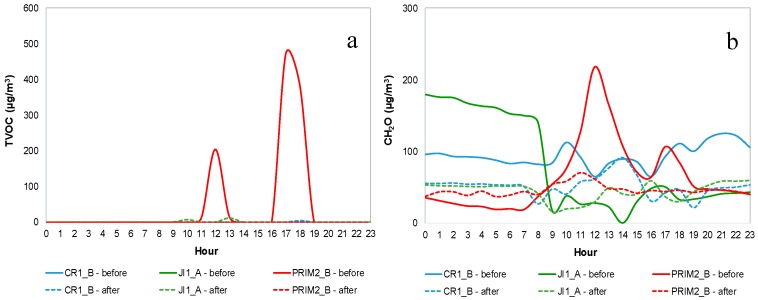
Mean daily profiles for CR1_B, JI1_A and PRIM2_B: (**a**) Total volatile organic compounds (TVOC) (**b**) Formaldehyde (CH_2_O).

Figure 4 shows TVOC and CH_2_O mean daily profiles for CR1_B, JI1_A and PRIM2_B (as example), during both IAQ campaigns, before mitigation measures (continuous line) and after mitigation measures (dashed line). Figures S8 and S9 (Supplementary Materials) show TVOC and CH_2_O mean daily profile, respectively, for both IAQ campaigns in all buildings.

Although it was not possible to establish a pattern for TVOC and CH_2_O concentrations along the day, peaks appeared to have occurred mostly on weekdays, during and immediately after the occupation periods. On the weekend the concentrations of both pollutants were relatively constant, and for TVOC they were very close to zero. In CR1_A, CR3_A and CR3_C it was possible to identify a similar pattern between the concentrations of CH_2_O and TVOC with a Spearman correlation coefficient between 0.717 and 0.836 (*p* < 0.05), suggesting that these two pollutants were emitted simultaneously by the same source, in accordance with Yang, et al. [33]. In CR1_A a peak concentration of both pollutants was detected during the occupation period, specifically during the sleeping/nap time, which seemed to be related to deep cleaning actions in the living and lunch rooms, while children were in the sleeping room (attached to the living and lunch rooms). In turn, CR3_A and CR3_C had higher concentrations of CH_2_O and TVOC over the whole period of occupation, possibly related to the infant’s hygiene.

In the class and lunch rooms of buildings 1 and 3 there were small variations of CH_2_O concentrations along the day, coincident with entrances and exits, which seemed to be related with emissions from moving the furniture (tables, chairs, cabinets). CH_2_O concentrations increased during night and dawn due to their accumulation (decreased ventilation), and slightly decreased during the morning (opening of the building).

In PRIM2_A CH_2_O concentrations were high even during non-occupation periods, possibly due to a continuous internal source of this pollutant, plus poor ventilation in those periods. The same happened in PRIM2_C, although with lower concentrations. That pattern might also reflect deep cleaning actions carried out in the whole building during the measurements at those two ME. The remaining ME had almost constant concentrations. Branco, et al. [21] identified furniture as the main responsible for the indoor concentrations of CH_2_O in school ME, although reported maximum values of CH_2_O were lower (204 µg/m^3^) than those found in the present study. In turn, Yang, et al. [33] also identified emission from construction and furnishing materials as a possible cause for the increase of CH_2_O concentrations. A recent study carried out by Bradman, et al. [58], in 40 early childhood education facilities in California serving children aged 6 years or less, also detected CH_2_O at all sampling sites with values lower (max = 48.8 µg/m^3^) than those measured in the present study.

Emissions from products used in the cleaning activities were responsible for TVOC concentrations at the end of the occupation period in all the classrooms of building 2, in PRIM1_A (only in the second campaign) and in CR3_A. A deep cleaning action with the use of bleach was also responsible for the peak of TVOC concentrations (1448 µg/m^3^) measured in the second campaign in PRIM2_C. In the first campaign, poor ventilation may have been one of the determining factors for the presence of this pollutant at night in classrooms of building 2. Cano, et al. [59] found lower concentrations (max = 920 µg/m^3^) of this pollutant in their study in Porto schools, while Branco, et al. [21] recorded TVOC concentration peaks (2320 µg/m^3^) in an urban nursery, higher than in the present study. A peak TVOC concentration was found in a lunch room (RF1) and in a sleeping room (CR3_D1) during the first campaign. In the first ME, there was an increase during and after the snack period, which is thought to be related to the post-meal cleaning activities, while in the second ME the peak concentration occurred during the occupation period, thus it seemed to be related to the hygiene of the children (diaper change) during that period. Mishra, et al. [31] studied the IAQ of 25 primary schools in Brisbane (Australia), having identified cleaning products as the main cause of indoor concentrations of VOC in those ME, followed by air fresheners and also art activities using glues and inks.

ME for infants and pre-schoolers had higher concentrations of TVOC and CH_2_O than primary schools. In general, internal sources such as emissions from furniture associated with lack of ventilation seemed to be the main responsible for the CH_2_O concentration, while cleaning actions seemed to be the main responsible for the presence of TVOC. Jovanović, et al. [34] and Nunes, et al. [23] reached the same conclusions. It is important to highlight the need for improvement in ventilation, as well as for a careful selection of materials and products used in these spaces—avoiding the use CH_2_O and VOC emitting materials and products.

#### 3.1.5. CO, NO_2_ and O_3_

**Figure 5:**
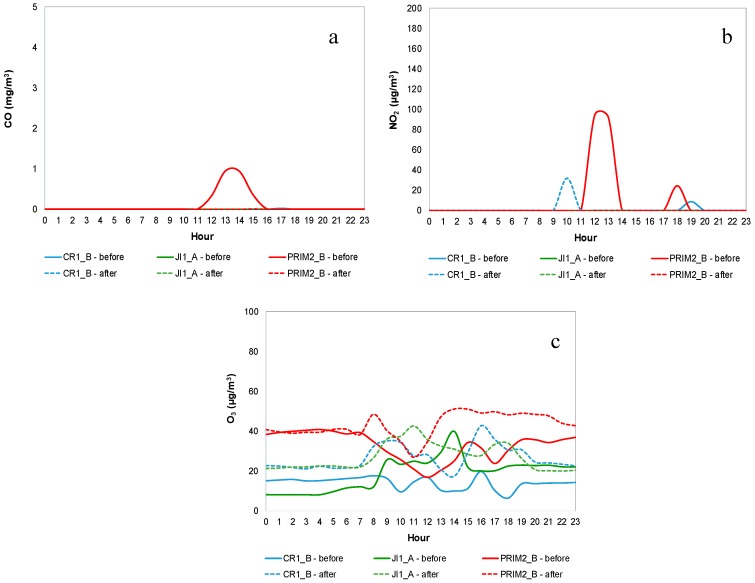
Mean daily profiles for CR1_B, JI1_A and PRIM2_B: (**a**) Carbone monoxide (**b**) Nitrogen dioxide (**c**) Ozone.

Figure 5 shows CO, NO_2_ and O_3_ mean daily profiles for CR1_B, JI1_A and PRIM2_B (as example), during both IAQ campaigns, before mitigation measures (continuous line) and after mitigation measures (dashed line). Figures S10–S12 (Supplementary Materials) show CO, NO_2_ and O_3_ mean daily profile, respectively, for both IAQ campaigns in all buildings.

It was possible to identify almost constant profiles of these pollutants during the non-occupation period and weekends, with lower concentrations when compared to the occupation periods—very close to zero for CO and NO_2_. Dorizas, et al. [56] reported similar results.

Regarding CO, it was possible to distinguish a similarity in the concentration pattern in almost all the studied ME on weekdays, characterized by: (i) a slight increase in the early morning; and (ii) a decrease in the late afternoon/early evening. In general, the highest concentrations were found in the lunch rooms—RF1 and RF3 (max = 3.51 mg/m^3^ and 5.13 mg/m^3^, respectively), possibly because this ME had higher influence from outdoor air intrusion (higher number of doors and windows directly to the outdoor of the building), and also because in these ME gas stoves were used during the period of occupation. Branco, et al. [21] reached the same conclusion, having identified outdoor air as a main cause for the indoor concentrations found in scholar ME, which were very similar to those found in the present study (max = 4.96 mg/m^3^).

Higher NO_2_ concentrations observed in lunch rooms appeared to have the same sources identified for CO. Vassura, et al. [30] stated that the presence of indoor sources for CO and NO_2_ was not expected in a preschool and an elementary school located in the suburban area of Bologna, Italy. Besides, it was possible to identify different mean daily profiles of NO_2_ among the various ME. In CR1_A, PRIM2_C, CR3_A and CR3_C, NO_2_ concentration profiles were coincident with the profiles presented for TVOC and CH_2_O. Cross-sensitivity between NO_2_ and the other sensors could be the explanation for that. 

Regarding O_3_ it was possible to identify relevant variations in its concentrations along the day in all the studied ME, mainly during the occupation period. The highest concentrations were recorded during the afternoon, with a maximum of 71.6 µg/m^3^ for PRIM1_B during lunch time in the second campaign. As far as known, there were no indoor sources of O_3_, so such patterns were probably caused by the intrusion of outdoor air through windows opening. The I/O ratio (0.07) from a study carried out in a primary school located in Zajecar (Serbia) showed the predominance of this pollutant in outdoor air [34]. On the other hand, and although several studies concluded that outdoor concentrations are usually found lower in urban than in rural and suburban environments [60,61], this was not observed in the indoor environments studied, because although buildings 2 and 3 were located in suburban areas and building 1 in an urban one, the last one did not evidence lower concentrations than the others, probably due to the air intrusion instabilities.

Thus, intrusion from outdoor air seemed to be the main source of CO, NO_2_ and O_3_ in the studied indoor ME. The same was concluded by Vassura, et al. [30], Branco, et al. [21] and Nunes, et al. [23].

#### 3.1.6. Radon

**Figure 6:**
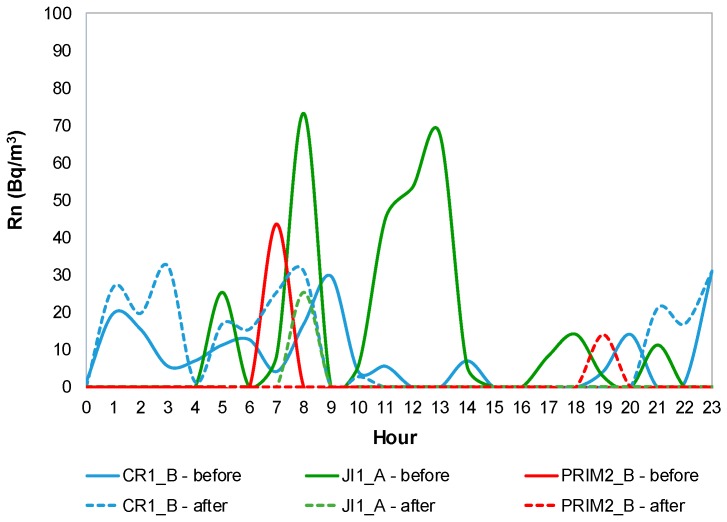
Radon mean daily profile for CR1_B, JI1_A and PRIM2_B.

Figure 6 shows Rn mean daily profile profiles for CR1_B, JI1_A and PRIM2_B (as example), during both IAQ campaigns, before mitigation measures (continuous line) and after mitigation measures (dashed line). Figures S13 (Supplementary Materials) shows radon mean daily profile for both IAQ campaigns in all buildings. 

A similar mean daily pattern of Rn was observed in all the studied ME, characterized by higher concentrations during weekends and non-occupation periods, followed by a decrease along the day on weekdays. As the main source of this pollutant has been usually associated with continuous release from soil and it enters the building through cracks and fissures in building foundations, it usually tended to accumulate in indoor ME during periods of closure, therefore not ventilated. In RF1 and PRIM2_C a peak concentration was detected during the afternoon and after the lunch time, respectively, which is thought to be associated with insufficient ventilation. Radon concentrations ranged from 0 Bq/m^3^ to 82 Bq/m^3^ (CR1_A), very similar to those reported by Kalimeri, et al. [62] in a study carried out in primary schools in Kozani, Greece (11 to 84 Bq/m^3^). In addition, Branco, et al. [43] reported similar mean concentrations of this pollutant in different school buildings in Porto (101 Bq/m^3^, 37 Bq/m^3^ and 57 Bq/m^3^, respectively for nursery schools for infants, nursery schools for pre-schoolers and primary schools). On the other hand, it would be expected that in the same building, ME on the ground floor would have higher concentrations than those on the floors above, but PRIM1_A and PRIM1_B, located on the first floor of building 1, had higher concentrations than the other ME on the ground floor of the same building. The infiltration of this pollutant, through other fissures, as well as possible different ventilation patterns that allowed reducing concentrations more effectively on the ground floor, could explain those unexpected results. In general, the difference in monitoring periods seems to be more noticeable in temperature and relative humidity’s results. Anyway, season and conditions should be the same to avoid biases in the results. 

### 3.2. Evaluation of the Mitigation Measures Implemented

Table 2 shows the specific IAP mitigation measures suggested and the status of application in each studied ME. From the five different types of possible IAP mitigation measures, only Types I and II (raising awareness and behavioural changes) were applied, simultaneously, and effects on concentrations of pollutants by type of measure could not be distinguished. It should be noted that this study was a preliminary approach where only low-cost measures were possible to put into practice, mostly, due to financial limitations.

The evaluation of the impact of the IAP mitigation measures implemented was carried out on the pollutants that exceeded at least one of the legislated/referenced values (Portuguese legislation or WHO), in the first IAQ campaign. Moreover, the second campaign confirmed that after the application of the IAP mitigation measures pollutants without exceedances in the first campaign maintained it in the second campaign (CO, TVOC and Rn).

As there were no reference values for PM_1_, TSP and O_3_, they were also not included. Since NO_2_ high concentrations registered seemed to be related to the cross-sensitivity with other sensors, this pollutant was also not included in the evaluation. Thus, Table 3, Table 4, Table 5 and Table 6 show mean and median concentrations, as well as the respective exceedances (%) to the reference values for the occupation period of PM_2.5_, PM_10_, CO_2_ and CH_2_O, respectively, as well as the respective *p*-value calculated on the evaluation of the difference between mean values in each IAQ campaign.

In the first campaign, PM_2.5_ and PM_10_ exceeded reference values both from WHO and Portuguese legislation in ME from all the three studied buildings. Regarding PM_2.5_ (Table 3) and PM_10_ (Table 4), WHO guideline was exceeded in 11 and 8 of 16 studied ME, respectively, while Portuguese limit was exceeded only in 6 and 2 of them, respectively. Worst results were found for finer than for coarser PM fraction. In fact, other authors reported the difficulty to achieve the restrictive reference values for PM_2.5_ [19,22,28,29], and finer particles are the most harmful for human health. The Portuguese legislated limits for both PM fractions were less restrictive due to the applied margin of tolerance (100%) because no mechanical ventilation was used. 

The second campaign was performed to evaluate the impact of the IAP mitigation measures applied in each studied ME (Table 2), by comparing results with those from the first campaign. With exception of PRIM1_B, PM_2.5_ concentrations decreased in all the studied ME of building 1. However, differences were only statistically significant (*p* = 0.029) in CR1_B. A positive improvement was found in 4 of the 6 ME, where PM_2.5_ concentrations decreased to levels below at least one of the reference values. For PM_10_ the number of ME above the reference levels decreased from 4 to only 1 in building 1, with statistically significant decreases in CR1_B (*p* = 0.013) and JI1_A (*p* = 0.006). In fact, in building 1, decreases were more significant in the classrooms for infants.

On the contrary, in most of the ME of buildings 2 and 3, PM_2.5_ and PM_10_ mean concentrations both increased, being some statistically significant (*p* < 0.05). Regarding building 2 not all the suggested IAP mitigation measures were applied. Moreover, the intensification of cleaning actions that was applied, but with inappropriate products (e.g., sweeping), due to financial constraints from the institution in acquiring more adequate and efficient cleaning material (e.g., vacuum cleaner), led to higher PM concentrations than in the first campaign. In building 3 most of the suggested IAP mitigation measures were applied in all ME. However, the increased number of occupants in the second campaign (due to a considerable number of absences in the first campaign) led to that increase in PM_2.5_ concentrations. CO_2_ concentrations in the first campaign (Table 5) were usually above the reference level in 5 of the 15 studied ME, which is commonly found in Portuguese nursery and primary schools [20,23,24].

Inadequate ventilation together with overcrowding in classrooms seemed to be responsible for those results. In fact, ASHRAE [51] recommends that occupational density in schools should not exceed 25 occupants per 100 m^2^, which was exceeded in all studied ME except for CR1_A. Portuguese legislation is less restrictive, since it is more focused on economic and educational criteria, defining the number of students per room of: (i) 10 children per room under 1 year old, 14 children per room between 1 and 2 years old, and 18 children per room between 2 and 3 years old [63]; (ii) 20 to 25 children per nursery room for pre-schoolers [64]; and (iii) 26 students per room from primary school [64]. It also defines a minimum area of 2 m^2^ per infant, between 1 and 2 years old, and the minimum area for each child besides 16 occupants is reduced to 1 m^2^ [63]. Although all the studied ME were in agreement with the Portuguese legislation for the number of occupants per classroom, exceedances of CO_2_ reference values occurred, which enhances the negative influence of inadequate ventilation and the need for a revision of the Portuguese legislation regarding this issue. In the second campaign, it was possible to notice a positive influence from the increase in ventilation. A decrease in the mean CO_2_ concentrations in all studied ME, except in CR3_D2 was observed. Statistically significant decreases (*p* < 0.005) were observed for CR1_B, JI1_A and PRIM2_B. Consequently only PRIM2_A maintained the number of exceedances. Thus, the low-cost and simple IAP mitigation measures implemented seemed to be enough to reduce CO_2_ concentrations to levels below the reference. A study carried out by Gao, et al. [65] concluded that even without mechanical ventilation, habits to open and close windows have a great impact on CO_2_ concentration, however this habit should be a temporary solution and a more definite solution should be applied [24].

A concerning situation was found for CH_2_O, in the first campaign, with a high number of exceedances to both Portuguese legislation and WHO reference values (the last is more restrictive).

In fact, nine of the 15 studied ME exceeded at least one of those references. In building 1, exceedances were found in the classrooms for infants (CR1_A and CR1_B), although in building 3 all the studied ME exceeded WHO reference value for CH_2_O. As the CH_2_O concentrations found were mainly due to the cleaning activities (as stated in Section 3.1.4), IAP mitigation measures suggested also focused those activities (Table 2). After the implementation of IAP mitigation measures, increases in CH_2_O concentrations were found in all the ME of building 2, while in buildings 1 and 3 there was a decrease in almost all the ME. Although in 3 ME CH_2_O concentrations decreased to levels below the reference values (CR1_B, CR3_B and CR3_D2), all of them in rooms occupied by infants, CH_2_O concentrations increased to levels over the reference values in other 2 ME (RF1 and PRIM2_C). Thus, IAP mitigation measures applied were not enough to obtain a significant reduction of the CH_2_O concentrations initially found to acceptable levels for occupants’ health protection.

In a global perspective, Type I and II IAP mitigation measures were possible to apply within all the studied ME. Although these types of IAP mitigation measures were the less expensive and the simplest to apply, they were enough to reduce IAP in all the studied ME, especially concerning CO_2_ concentrations. For PM_2.5_ and PM_10_ those measures presented also good results, although other IAP mitigation measures should be applied to further reduce the concentrations. For CH_2_O, results were poor, so other types of IAP mitigation measures (more expensive and more complex) need to be applied.

## 4. Conclusions

This study allowed to evaluate the low-cost IAP mitigation measures implemented in nursery and primary schools. A first IAQ campaign in different ME (classrooms, lunch rooms and sleeping rooms) allowed to identify that the major IAP problems were associated to high levels of CO_2_, PM (especially finer fractions), and CH_2_O. Concentrations of those pollutants were above the reference values of both Portuguese legislation and WHO. Other pollutants, namely CO, NO_2_, O_3_, TVOC and Rn did not present concerning situations in the studied ME. The high number of children (occupation) and their usual activities, inadequate ventilation habits, cleaning activities, use of products and materials emitting CH_2_O, as well as some intrinsic characteristics of the building were the main sources for those problems. 

Several measures were implemented aiming to mitigate those major IAP problems. Due to financial limitations, it was only possible to implement, simultaneously, Types I and II IAP mitigation measures (raising awareness and behavioural changes) in all the studied ME. Although these were the less expensive and the simplest to apply, they resulted in the reduction of IAP in all the studied ME, without affecting negatively the pollutants concentration that did not present concerning situations in the first campaign. Effective reductions were achieved in CO_2_ concentrations, while in PM_2.5_ and PM_10_ other IAP mitigation measures should be implemented in some ME to achieve a more effective reduction in concentrations, especially in finer fractions. The low-cost IAP mitigation measures implemented did not decrease CH_2_O concentrations to below the reference values for health protection, thus more expensive and complex measures need to be implemented. 

Due to the usual IAP problems in nursery and primary schools, intervention studies are needed. Thus, the evaluation methodology developed in the present study emerges as a useful tool for these kind of studies. For the future, the application of more expensive and complex IAP mitigation measures should be evaluated. In other hand, to validate this methodological approach, more tests should be performed, and the type of measure should be studied one by one to understand the effect of each one in IAQ. It would be also important to follow up the implementation of these measures by evaluating their impact longitudinally. To reproduce this type of study in other contexts it is recommended that the IAQ characterization period should not be too large and the conditions should be the same.

Besides, a more extensive and comprehensive study is recommended in order to provide a strong and quantifiable comparison between all the low-cost mitigation measures to improve air quality and for their real, economic and practical implementation, as well as their effects on the energy sustainability, thermal comfort (e.g., PMV and PPD), health and security of the occupants (children and school staff).

## Figures and Tables

**Table 1:** Main characteristics of each studied microenvironment.

ME	Use	Class/Grade ^a^	Floor ^b^	Area (m^2^)	Occupancy (Children + Staff)	Period of Occupation
CR1_A	Classroom	<1 year	GF	48	10 + 3	9:30–18:00 11:45–14:00 ^c^
CR1_B	Classroom	2 years	GF	40	19 + 3	9:00–11:30 13:00–19:00 13:00–15:30 ^c^
JI1_A	Classroom	4 years	GF	50	26 + 2	9:30–12:00 13:30–18:00
PRIM1_A	Classroom	1st grade	1st	48	25 + 1	9:00–13:00 14:30–16:30
PRIM1_B	Classroom	4th grade	1st	53	20 + 1	9:00–13:00 14:30–16:30
RF1	Lunch room	1 year–4th grade	GF (back)	92	68 to 100	11:00–14:00
JI2_A	Classroom	Mixed (3–5 years)	GF	55	25 + 2	9:00–12:00 13:30–15:30
PRIM2_A	Classroom	1st grade	GF	55	20 + 1	9:00–12:30 14:00–17:30
PRIM2_B	Classroom	2nd grade	GF	55	26 + 1	9:00–12:30 14:00–17:30
PRIM2_C	Classroom	3rd grade	GF	55	22 + 1	9:00–12:30 14:00–17:30
CR3_A	Classroom	1 year	1st	36	14 + 2	7:30–10:00 11:30–19:00 12:00–15:00 ^c^
CR3_B	Classroom	2 years	1st	39	18 + 2	9:00–11:45
CR3_C	Classroom	Mixed (1–2 years)	1st	51	15 + 2	9:00–10:45
CR3_D1	Sleeping room	2 years	1st	38	18 + 2	12:00–15:00 ^c^
CR3_D2	Sleeping room	Mixed (1–2 years)	1st	36	15 + 2	12:00–15:00 ^c^
RF3	Lunch room	1–5 years	1st	148	14 to 64	10:30–12:30

^a^ 1st grade—children aged 6–7 years; 2nd grade—children aged 7–8 years; 3rd grade—children aged 8–9 years; 4th grade—children aged 9–10 years; ^b^ 1st—first floor; GF—ground floor; ^c^ sleeping time.

**Table 2:** IAP mitigation measures and its application in the studied ME.

Type	Measure	CR1		JI1	PRIM1		RF1	JI2	PRIM2			CR3					RF3
		A	B	A	A	B		A	A	B	C	A	B	C	D1	D2	
I	Awareness raising of the coordinators of the schools, teachers, collaborators and students, about the importance and influence of the IAQ in schools and children. Education about good practices of ventilation, cleaning and hygiene, characteristics of certain cleaning products and materials used in handwork (glues, paints) could positively influence behaviour and lead to improved health	**☑**	**☑**	**☑**	**☑**	**☑**	**☑**	**☑**	**☑**	**☑**	**☑**	**☑**	**☑**	**☑**	**☑**	**☑**	**☑**
II	Open the windows to the outdoor and the door to the inner corridor before the occupation period	**⊠**	**☑**	**☑**	**☑**	**⊠**	**☑**	**☑**	**⊠**	**☑**	**☑**	**☑**	**☑**	**☑**	**☑**	**☑**	**☑**
	Semi-open windows to the outdoor and doors to the inner corridor during the occupation period	**☑**	**☑**	**☑**	**☑**	**☑**	**☑**	**⊠**	**⊠**	**⊠**	**⊠**	**☑**	**☑**	**☑**	**☑**	**☑**	**☑**
	Open the windows to the outdoor during the painting/collage activities	**☐**	**☑**	**☐**	**☐**	**☐**	**☐**	**☑**	**☑**	**☑**	**☐**	**☐**	**☑**	**☐**	**☐**	**☐**	**☐**
	Open the windows to the outdoor and the door to the inner corridor during the lunch and breaks	**☑**	**☑**	**☑**	**☑**	**☑**	**☑**	**⊠**	**⊠**	**⊠**	**⊠**	**☑**	**☐**	**☐**	**☐**	**☐**	**☐**
	Open the windows to the outdoor and the door to the inner corridor during and after the cleaning activities	**⊠**	**☑**	**⊠**	**⊠**	**⊠**	**☑**	**⊠**	**⊠**	**⊠**	**☑**	**☑**	**☑**	**☑**	**☐**	**☑**	**☑**
	Leave the door to the inner corridor open at night	**⊠**	**⊠**	**⊠**	**⊠**	**⊠**	**☑**	**☑**	**⊠**	**☑**	**☑**	**☑**	**⊠**	**☑**	**☑**	**☑**	**☑**
	Improve the cleaning using a vacuum cleaner, damp cloth and utensils and electrostatic products after occupation period	**⊠**	**☑**	**⊠**	**⊠**	**⊠**	**☑**	**⊠**	**⊠**	**⊠**	**⊠**	**☑**	**☑**	**☑**	**☑**	**☑**	**☑**
III	Replace the broom by the vacuum cleaner or electrostatic utensils in cleaning activities	**☐**	**☐**	**☐**	**☐**	**☐**	**⊠**	**⊠**	**⊠**	**⊠**	**⊠**	**☐**	**☐**	**☐**	**☐**	**☐**	**☐**
	Use of different rooms, depending on the type of activity carried out by the children	**☐**	**☐**	**☐**	**☐**	**☐**	**☐**	**☐**	**☐**	**☐**	**☐**	**⊠**	**☐**	**☐**	**☐**	**☐**	**☐**
IV	Use the electric heating during the occupation period	**☐**	**☐**	**☐**	**☐**	**☐**	**☐**	**⊠**	**⊠**	**⊠**	**⊠**	**☐**	**☐**	**☐**	**☐**	**☐**	**☐**
V	Replace the existing chalkboard by another to avoid the emission of PM (e.g., whiteboard)	**☐**	**☐**	**☐**	**☐**	**☐**	**☐**	**☐**	**☐**	**☐**	**⊠**	**☐**	**☐**	**☐**	**☐**	**☐**	**☐**
	Install a mechanical ventilation system or build windows	**☐**	**☐**	**☐**	**☐**	**☐**	**☐**	**☐**	**☐**	**☐**	**☐**	**☐**	**☐**	**☐**	**⊠**	**☐**	**☐**

**☑**—IAP mitigation measures suggested and applied; **⊠**—IAP mitigation measures suggested but not applied; **☐**—IAP mitigation measures not suggested.

**Table 3:** Average, median, exceedances to the legislation and the *p* value of the difference between the hourly mean before and after implementation of IAP mitigation measure for the occupation period for PM_2.5_.

ME	(PM_2.5_)_in_^a^						(PM_2.5_)_post_^b^						*p* Value
	Avg ^c^	Med ^d^	Exceedances (%)				Avg ^c^	Med ^d^	Exceedances (%)				
			WHO ^e^		PL ^f^				WHO ^e^		PL ^f^		
CR1_A	33.22	31.25	100	**✕**	0	**✓**	28.58	28.75	0	**✓**	0	**✓**	0.157
CR1_B	44.58	43.63	33	**✕**	50	**✕**	34.91	33.00	67	**✕**	0	**✓**	0.029 *
JI1_A	68.94	58.63	33	**✕**	100	**✕**	50.23	47.38	67	**✕**	50	**✕**	0.136
PRIM1_A	34.07	34.17	50	**✕**	0	**✓**	31.03	35.88	0	**✓**	0	**✓**	0.713
PRIM1_B	30.01	31.06	75	**✕**	0	**✓**	35.30	34.56	33	**✕**	50	**✕**	0.442
RF1	38.29	42.50	100	**✕**	50	**✕**	21.21	21.38	0	**✓**	0	**✓**	0.100
JI2_A	34.14	32.13	0	**✓**	0	**✓**	109.21	102.75	67	**✕**	100	**✕**	**<0.001**
PRIM2_A	32.00	33.00	100	**✕**	0	**✓**	36.38	36.75	50	**✕**	0	**✓**	0.233
PRIM2_B	20.42	19.75	0	**✓**	0	**✓**	24.11	23.38	0	**✓**	0	**✓**	0.310
PRIM2_C	28.94	23.75	100	**✕**	0	**✓**	34.56	30.00	0	**✓**	0	**✓**	0.233
CR3_A	37.05	35.63	67	**✕**	50	**✕**	54.74	53.13	33	**✕**	50	**✕**	**0.006**
CR3_B	30.67	32.25	0	**✓**	50	**✕**	22.67	22.50	0	**✓**	0	**✓**	0.400
CR3_C	28.13	28.13	0	**✓**	0	**✓**	120.83	120.83	100	**✕**	100	**✕**	0.333
CR3_D1	29.83	25.00	50	**✕**	0	**✓**	23.13	20.00	0	**✓**	0	**✓**	0.400
CR3_D2	18.25	14.25	0	**✓**	0	**✓**	29.21	25.25	33	**✕**	0	**✓**	0.400
RF3	853.42	894.50	100	**✕**	100	**✕**	19.92	20.75	50	**✕**	0	**✓**	0.100

**^a^** PM_2.5_ concentration of first IAQ campaign; **^b^** PM_2.5_ concentration of second IAQ campaign (after IAP mitigation measures implemented); **^c^** Average; **^d^** Median; **^e^** % of exceedances to the World Health Organization (WHO); **^f^** % of exceedances to the Portuguese legislation; * *p* < 0.05 (considered statistically significant) for the item in bold; **✕**—Exceedances to the Portuguese legislation or WHO; **✓**—No exceedances to the Portuguese legislation or WHO.

**Table 4:** Average, median, exceedances to the legislation and the *p* value of the difference between the hourly mean before and after implementation of IAP mitigation measure for the occupation period for PM_10_.

ME	(PM_10_)_in_^a^						(PM_10_)_post_^b^						*p* Value
	Avg ^c^	Med ^d^	Exceedances (%)				Avg ^c^	Med ^d^	Exceedances (%)				
			WHO ^e^		PL ^f^				WHO ^e^		PL ^f^		
CR1_A	43.06	39.25	0	**✓**	0	**✓**	41.72	40.75	0	**✓**	0	**✓**	0.757
CR1_B	71.95	70.25	33	**✕**	0	**✓**	50.40	47.63	0	**✓**	0	**✓**	0.013 *
JI1_A	120.44	103.00	33	**✕**	100	**✕**	63.58	61.63	33	**✕**	0	**✓**	**0.006**
PRIM1_A	54.80	51.67	25	**✕**	0	**✓**	53.81	62.63	0	**✓**	0	**✓**	0.959
PRIM1_B	38.03	38.85	0	**✓**	0	**✓**	40.42	38.13	0	**✓**	0	**✓**	0.563
RF1	47.04	51.25	33	**✕**	0	**✓**	29.33	29.75	0	**✓**	0	**✓**	0.100
JI2_A	64.18	59.25	0	**✓**	0	**✓**	140.57	122.75	67	**✕**	100	**✕**	**0.011**
PRIM2_A	53.67	54.75	0	**✓**	0	**✓**	60.99	59.38	0	**✓**	0	**✓**	0.480
PRIM2_B	33.61	31.25	0	**✓**	0	**✓**	33.76	32.25	0	**✓**	0	**✓**	0.931
PRIM2_C	55.81	40.75	100	**✕**	0	**✓**	54.36	47.75	0	**✓**	0	**✓**	0.605
CR3_A	43.88	42.38	67	**✕**	0	**✓**	62.01	58.25	33	**✕**	50	**✕**	**0.010**
CR3_B	37.33	42.50	0	**✓**	0	**✓**	28.79	28.75	0	**✓**	0	**✓**	0.400
CR3_C	36.13	36.13	0	**✓**	0	**✓**	136.69	136.69	100	**✕**	50	**✕**	0.333
CR3_D1	37.08	29.13	50	**✕**	0	**✓**	26.00	21.50	0	**✓**	0	**✓**	0.400
CR3_D2	23.17	16.75	0	**✓**	0	**✓**	35.63	29.00	0	**✓**	0	**✓**	0.400
RF3	860.25	903.25	100	**✕**	100	**✕**	23.50	25.50	0	**✓**	0	**✓**	0.100

**^a^** PM_10_ concentration of first IAQ campaign; **^b^** PM_10_ concentration of second IAQ campaign (after IAP mitigation measures implemented); **^c^** Average; **^d^** Median; **^e^** % of exceedances to the World Health Organization (WHO); **^f^** % of exceedances to the Portuguese legislation; * *p* < 0.05 (considered statistically significant) for the item in bold; **✕**—Exceedances to the Portuguese legislation or WHO; **✓**—No exceedances to the Portuguese legislation or WHO.

**Table 5:** Average, median, exceedances to the legislation and the *p* value of the difference between the hourly mean before and after implementation of IAP mitigation measure for the occupation period for CO_2_.

ME	(CO_2_)_in_^a^				(CO_2_)_post_^b^				*p* Value
	Avg ^c^	Med ^d^	Exceedances (%)		Avg ^c^	Med ^d^	Exceedances (%)		
			PL ^e^				PL ^e^		
CR1_A	2543	2522	0	**✓**	2354	2201	0	**✓**	0.489
CR1_B	3745	4008	100	**✕**	1840	1350	0	**✓**	0.002 *
JI1_A	2764	2847	100	**✕**	1137	1155	0	**✓**	**0.001**
PRIM1_A	2175	2398	0	**✓**	1394	1452	0	**✓**	0.065
PRIM1_B	1709	1496	0	**✓**	966	910	0	**✓**	0.083
RF1	1402	1573	0	**✓**	1303	1321	0	**✓**	0.700
JI2_A	2635	2249	0	**✓**	1802	1614	0	**✓**	0.165
PRIM2_A	3300	3285	100	**✕**	3154	3454	100	**✕**	1.000
PRIM2_B	2761	2616	0	**✓**	1264	1011	0	**✓**	**<0.001**
PRIM2_C	4008	4464	100	**✕**	2793	2990	0	**✓**	0.077
CR3_A	1723	1719	0	**✓**	1503	1162	0	**✓**	0.291
CR3_B	1746	1649	0	**✓**	1176	1338	0	**✓**	0.100
CR3_D1	3161	3227	50	**✕**	2986	3089	0	**✓**	0.700
CR3_D2	2062	2175	0	**✓**	2259	2282	0	**✓**	0.400
RF3	2212	2162	0	**✓**	1249	1321	0	**✓**	0.100

**^a^** CO_2_ concentration of first IAQ campaign; **^b^** CO_2_ concentration of second IAQ campaign (after IAP mitigation measures implemented); **^c^** Average; **^d^** Median; **^e^** % of exceedances to the Portuguese legislation; * *p* < 0.05 (considered statistically significant) for the item in bold; **✕**—Exceedances to the Portuguese legislation or WHO; **✓**—No exceedances to the Portuguese legislation or WHO.

**Table 6:** Average, median, exceedances to the legislation and the *p* value of the difference between the hourly mean before and after implementation of IAP mitigation measure for the occupation period for CH_2_O.

ME	(CH_2_O)_in_^a^						(CH_2_O)_post_^b^						*p* Value
	Avg ^c^	Med ^d^	Exceedances (%)				Avg ^c^	Med ^d^	Exceedances (%)				
			WHO ^e^		PL ^f^				WHO ^e^		PL ^f^		
CR1_A	134.82	90,83	41	**✕**	100	**✕**	129.36	105.47	59	**✕**	100	**✕**	0.730
CR1_B	88.04	87.52	28	**✕**	50	**✕**	56.03	52.45	0	**✓**	0	**✓**	0.002 *
JI1_A	28.71	27.62	0	**✓**	0	**✓**	34.76	36.77	0	**✓**	0	**✓**	0.605
PRIM1_A	35.82	41.70	0	**✓**	0	**✓**	36.92	40.09	0	**✓**	0	**✓**	0.959
PRIM1_B	21.37	18.56	0	**✓**	0	**✓**	9.57	5.27	0	**✓**	0	**✓**	0.169
RF1	20.09	15.89	0	**✓**	0	**✓**	49.05	46.68	8	**✕**	0	**✓**	0.100
JI2_A	63.74	65.43	0	**✓**	0	**✓**	71.87	74.83	0	**✓**	0	**✓**	0.073
PRIM2_A	80.57	76.55	18	**✕**	0	**✓**	688.37	757.95	100	**✕**	100	**✕**	**<0.001**
PRIM2_B	109.74	106.25	41	**✕**	100	**✕**	52.61	48.50	3	**✕**	0	**✓**	**<0.001**
PRIM2_C	82.11	79.01	0	**✓**	0	**✓**	102.46	87.93	35	**✕**	100	**✕**	0.931
CR3_A	277.70	282.23	80	**✕**	100	**✕**	91.51	73.90	20	**✕**	100	**✕**	**<0.001**
CR3_B	190.21	192.82	91	**✕**	100	**✕**	33.49	28.13	0	**✓**	0	**✓**	0.100
CR3_D1	87.47	91.24	17	**✕**	0	**✓**	82.61	87.59	33	**✕**	0	**✓**	0.700
CR3_D2	85.32	76.10	21	**✕**	50	**✕**	49.31	48.76	0	**✓**	0	**✓**	0.100
RF3	133.64	144.41	100	**✕**	100	**✕**	74.51	77.03	25	**✕**	0	**✓**	0.100

**^a^** CH_2_O concentration of first IAQ campaign; **^b^** CH_2_O concentration of second IAQ campaign (after IAP mitigation measures implemented); **^c^** Average; **^d^** Median; **^e^** % of exceedances to the World Health Organization (WHO); **^f^** % of exceedances to the Portuguese legislation; * *p* < 0.05 (considered statistically significant) for the item in bold; **✕**—Exceedances to the Portuguese legislation or WHO; **✓**—No exceedances to the Portuguese legislation or WHO.

## Supplementary Materials

The following are available online at www.mdpi.com/1660-4601/14/6/585/s1, Figure S1: Temperature mean daily profile for: (a) Building 1—first campaign; (b) Building 1—second campaign (after IAP mitigation measures implemented); (c) Building 2—first campaign; (d) Building 2—second campaign (after IAP mitigation measures implemented); (e) Building 3—first campaign, (f) Building 3—second campaign (after IAP mitigation measures implemented), Figure S2: Relative Humidity mean daily profile for: (a) Building 1—first campaign; (b) Building 1—second campaign (after IAP mitigation measures implemented); (c) Building 2—first campaign; (d) Building 2—second campaign (after IAP mitigation measures implemented); (e) Building 3—first campaign, (f) Building 3—second campaign (after IAP mitigation measures implemented), Figure S3: PM_1_ mean daily profile for: (a) Building 1—first campaign; (b) Building 1—second campaign (after IAP mitigation measures implemented); (c) Building 2—first campaign; (d) Building 2—second campaign (after IAP mitigation measures implemented); (e) Building 3—first campaign, (f) Building 3—second campaign (after IAP mitigation measures implemented), Figure S4: PM_2.5_ mean daily profile for: (a) Building 1—first campaign; (b) Building 1—second campaign (after IAP mitigation measures implemented); (c) Building 2—first campaign, (d) Building 2—second campaign (after IAP mitigation measures implemented); (e) Building 3—first campaign, (f) Building 3—second campaign (after IAP mitigation measures implemented), Figure S5: PM_10_ mean daily profile for: (a) Building 1—first campaign; (b) Building 1—second campaign (after IAP mitigation measures implemented); (c) Building 2—first campaign, (d) Building 2—second campaign (after IAP mitigation measures implemented); (e) Building 3—first campaign, (f) Building 3—second campaign (after IAP mitigation measures implemented), Figure S6: TSP mean daily profile for: (a) Building 1—first campaign; (b) Building 1—second campaign (after IAP mitigation measures implemented); (c) Building 2—first campaign, (d) Building 2—second campaign (after IAP mitigation measures implemented); (e) Building 3—first campaign, (f) Building 3—second campaign (after IAP mitigation measures implemented), Figure S7: CO_2_ mean daily profile for: (a) Building 1—first campaign; (b) Building 1—second campaign (after IAP mitigation measures implemented); (c) Building 2—first campaign; (d) Building 2—second campaign (after IAP mitigation measures implemented); (e) Building 3—first campaign, (f) Building 3—second campaign (after IAP mitigation measures implemented), Figure S8: Formaldehyde mean daily profile for: (a) Building 1—first campaign; (b) Building 1—second campaign (after IAP mitigation measures implemented); (c) Building 2—first campaign; (d) Building 2—second campaign (after IAP mitigation measures implemented); (e) Building 3—first campaign, (f) Building 3—second campaign (after IAP mitigation measures implemented), Figure S9: Total organic volatile compounds mean daily profile for: (a) Building 1—first campaign; (b) Building 1—second campaign (after IAP mitigation measures implemented); (c) Building 2—first campaign; (d) Building 2—second campaign (after IAP mitigation measures implemented); (e) Building 3—first campaign, (f) Building 3—second campaign (after IAP mitigation measures implemented), Figure S10: Carbone monoxide mean daily profile for: (a) Building 1—first campaign; (b) Building 1—second campaign (after IAP mitigation measures implemented); (c) Building 2—first campaign; (d) Building 2—second campaign (after IAP mitigation measures implemented); (e) Building 3—first campaign, (f) Building 3—second campaign (after IAP mitigation measures implemented), Figure S11: Nitrogen dioxide mean daily profile for: (a) Building 1—first campaign; (b) Building 1—second campaign (after IAP mitigation measures implemented); (c) Building 2—first campaign; (d) Building 2—second campaign (after IAP mitigation measures implemented); (e) Building 3—first campaign, (f) Building 3—second campaign (after IAP mitigation measures implemented), Figure S12: Ozone mean daily profile for: (a) Building 1—first campaign; (b) Building 1—second campaign (after IAP mitigation measures implemented); (c) Building 2—first campaign; (d) Building 2—second campaign (after IAP mitigation measures implemented); (e) Building 3—first campaign, (f) Building 3—second campaign (after IAP mitigation measures implemented), Figure S13: Radon mean daily profile for: (a) Building 1—first campaign; (b) Building 1—second campaign (after IAP mitigation measures implemented); (c) Building 2—first campaign; (d) Building 2—second campaign (after IAP mitigation measures implemented); (e) Building 3—first campaign, (f) Building 3—second campaign (after IAP mitigation measures implemented), Table S1: Type of the suggested IAP mitigation measures and respective specification.
