# Supplementary material for: Evaluation of Low-Cost Mitigation Measures Implemented to Improve Air Quality in Nursery and Primary Schools
Source: Int J Environ Res Public Health. 2017 May 31;14(6):585. doi: 10.3390/ijerph14060585 (PMC5486271; doi:10.3390/ijerph14060585)
Supplement: Supplementary file 1 [file ijerph-14-00585-s001.pdf]

Supplementary Material

Table S1. Type of the suggested IAP mitigation measures and respective specification.

Type	Measure	Specification
**I**	Raising Awareness	Awareness raising of the scholar community, in particular, of the coordinators of the facilities, teachers and collaborators, and in some cases, even the students, about the importance and influence of the IAQ in schools and children. Education about good practices of ventilation, cleaning and hygiene, characteristics of certain cleaning products and materials used in handwork (glues, paints) could positively influence behaviour and lead to improved health. This measure serves as a basis for the implementation of the others.
**II**	Behavioural Changes	(i) Increasing ventilation: dilution, transport and dispersion of the pollutants present in the indoor air essentially through the systematic opening of doors and windows to the outdoor and to the interior corridors during the periods of no occupation, cleanings, activities of collages/ paintings and whenever possible also during the occupation.<br>(ii) Improving cleaning actions: cleaning the particles of surfaces and floors more carefully, favouring the use of vacuum cleaners, electrostatic utensils and/or damp cloths.
**III**	Changes of Products/ Materials and Places of Activities	(i) Replacement of floor cleaning with a broom by materials/ products mentioned in Type II measures.<br>ii) Use of different rooms, depending on the type of activity to be carried out (reception, sleeping, eating, prolongation).
**IV**	Technical and Technological Changes	(i) Use of equipment that allows reducing relative humidity (e.g. dehumidifier).<br>(ii) Optimization of the existing heating system or use solar radiation exposure to increase temperature.
**V**	Structural Changes	Replacing the existing chalkboards with different types of boards to avoid the emission of PM (e.g., whiteboard).

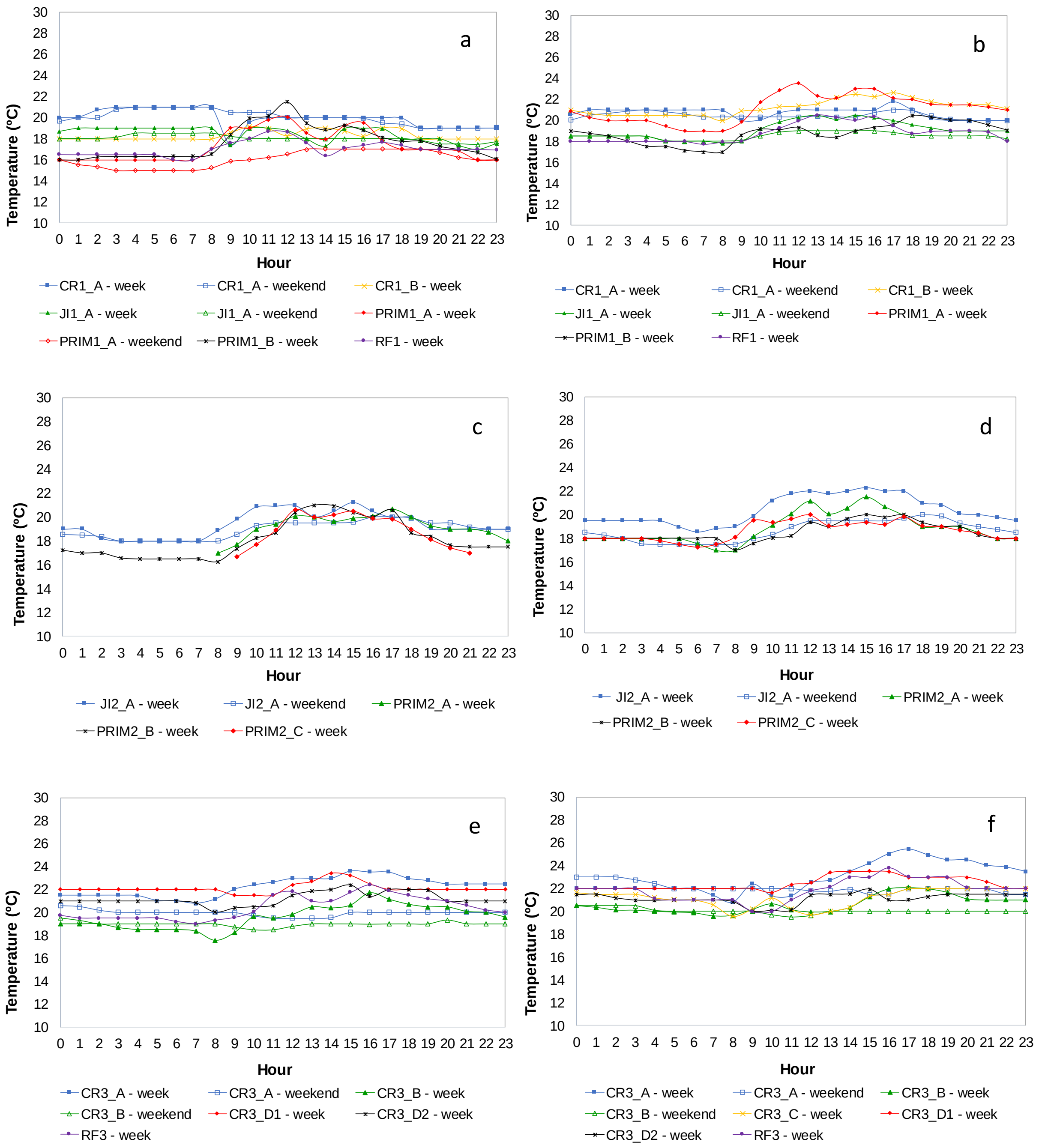

Figure S1. Temperature mean daily profile for: (**a**) Building 1—first campaign; (**b**) Building 1—second campaign (after IAP mitigation measures implemented); (**c**) Building 2—first campaign; (**d**) Building 2—second campaign (after IAP mitigation measures implemented); (**e**) Building 3—first campaign; (**f**) Building 3—second campaign (after IAP mitigation measures implemented).

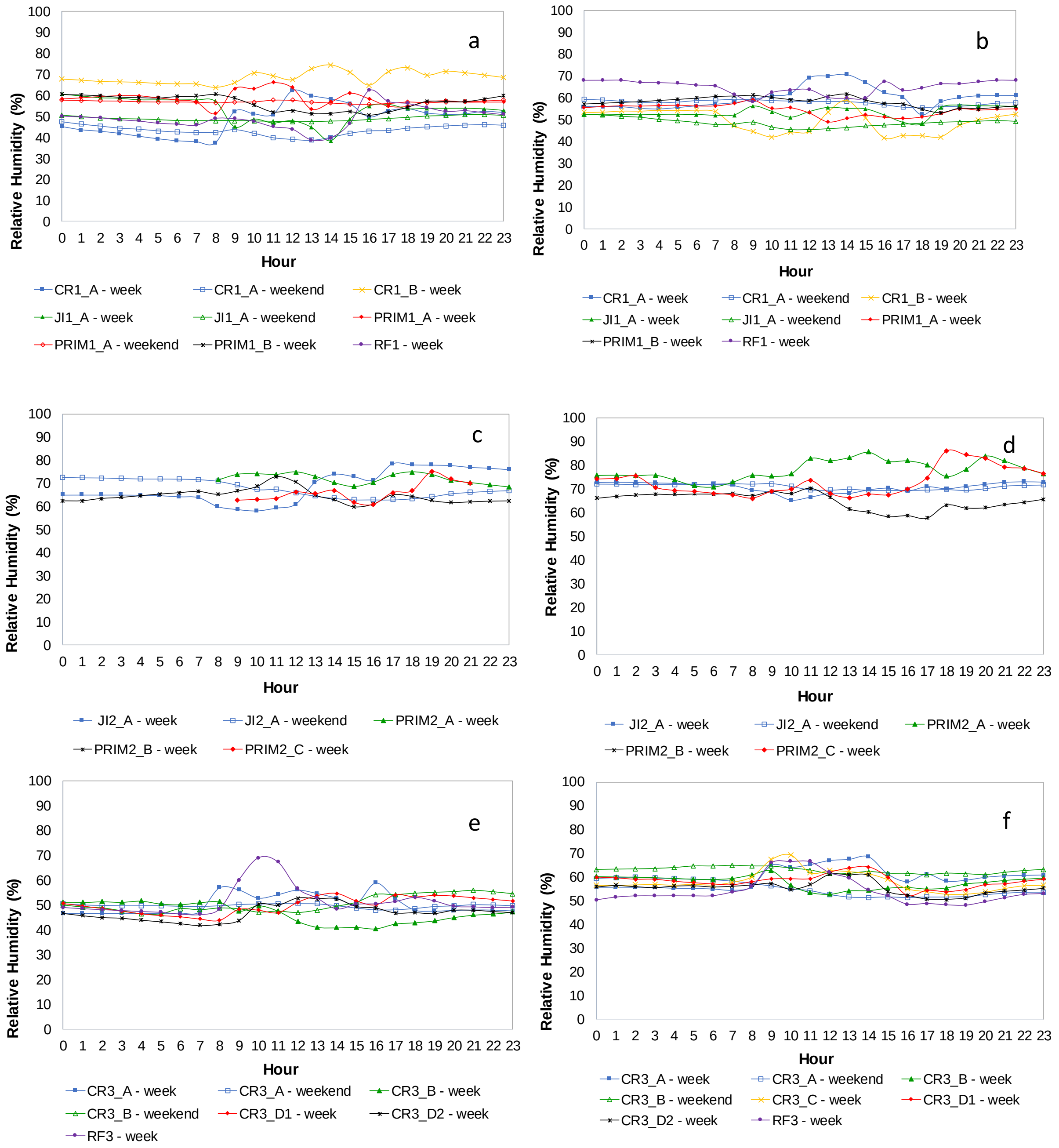

Figure S2. Relative Humidity mean daily profile for: (**a**) Building 1—first campaign; (**b**) Building 1—second campaign (after IAP mitigation measures implemented); (**c**) Building 2—first campaign; (**d**) Building 2—second campaign (after IAP mitigation measures implemented); (**e**) Building 3—first campaign, (**f**) Building 3—second campaign (after IAP mitigation measures implemented).

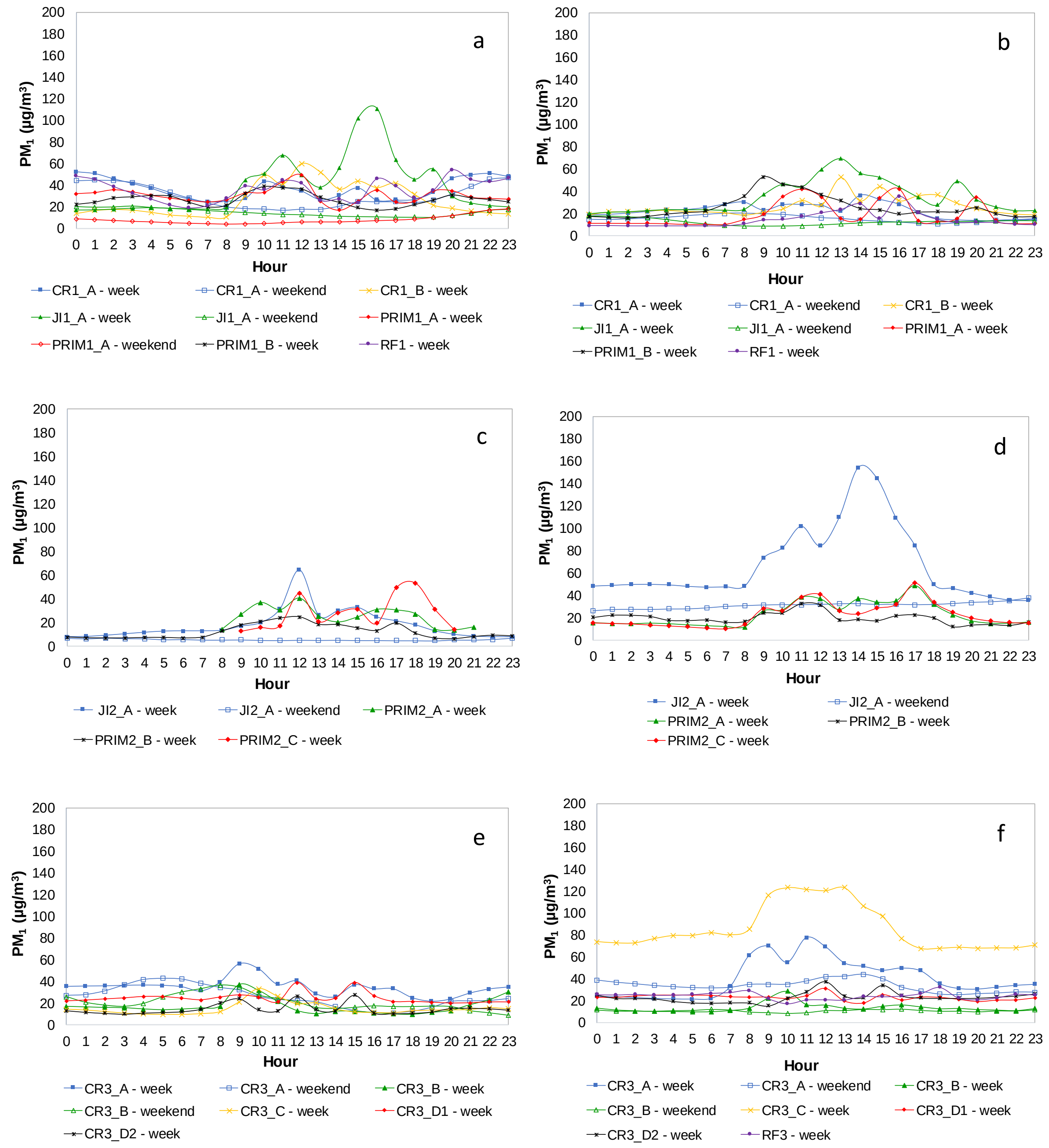

Figure S3. PM_1 mean daily profile for: (**a**) Building 1—first campaign; (**b**) Building 1—second campaign (after IAP mitigation measures implemented); (**c**) Building 2—first campaign, (**d**) Building 2—second campaign (after IAP mitigation measures implemented); (**e**) Building 3—first campaign, (**f**) Building 3—second campaign (after IAP mitigation measures implemented)

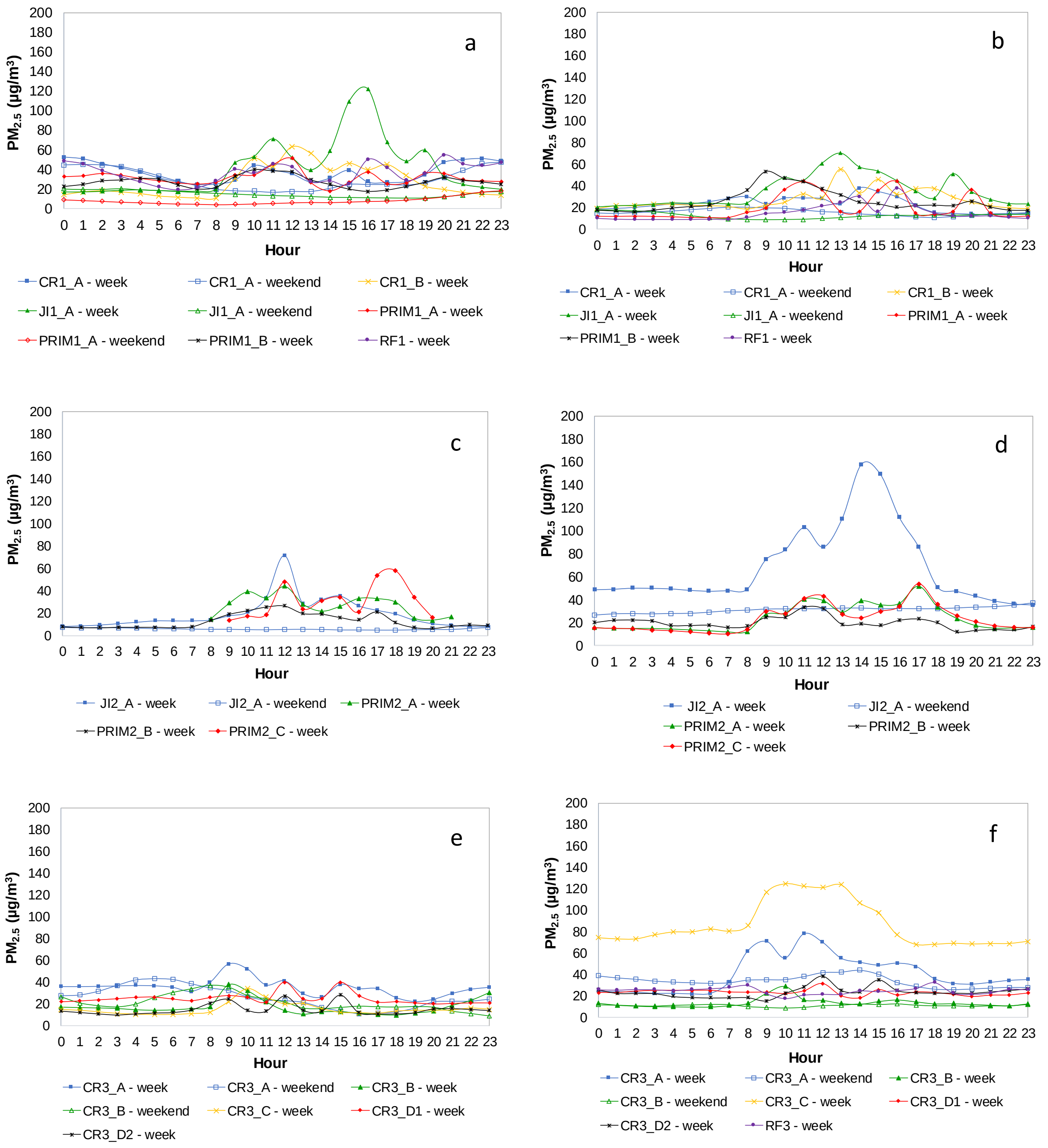

Figure S4. $PM_{2.5}$ mean daily profile for: (**a**) Building 1—first campaign; (**b**) Building 1—second campaign (after IAP mitigation measures implemented); (**c**) Building 2—first campaign, (**d**) Building 2—second campaign (after IAP mitigation measures implemented); (**e**) Building 3—first campaign, (**f**) Building 3—second campaign (after IAP mitigation measures implemented).

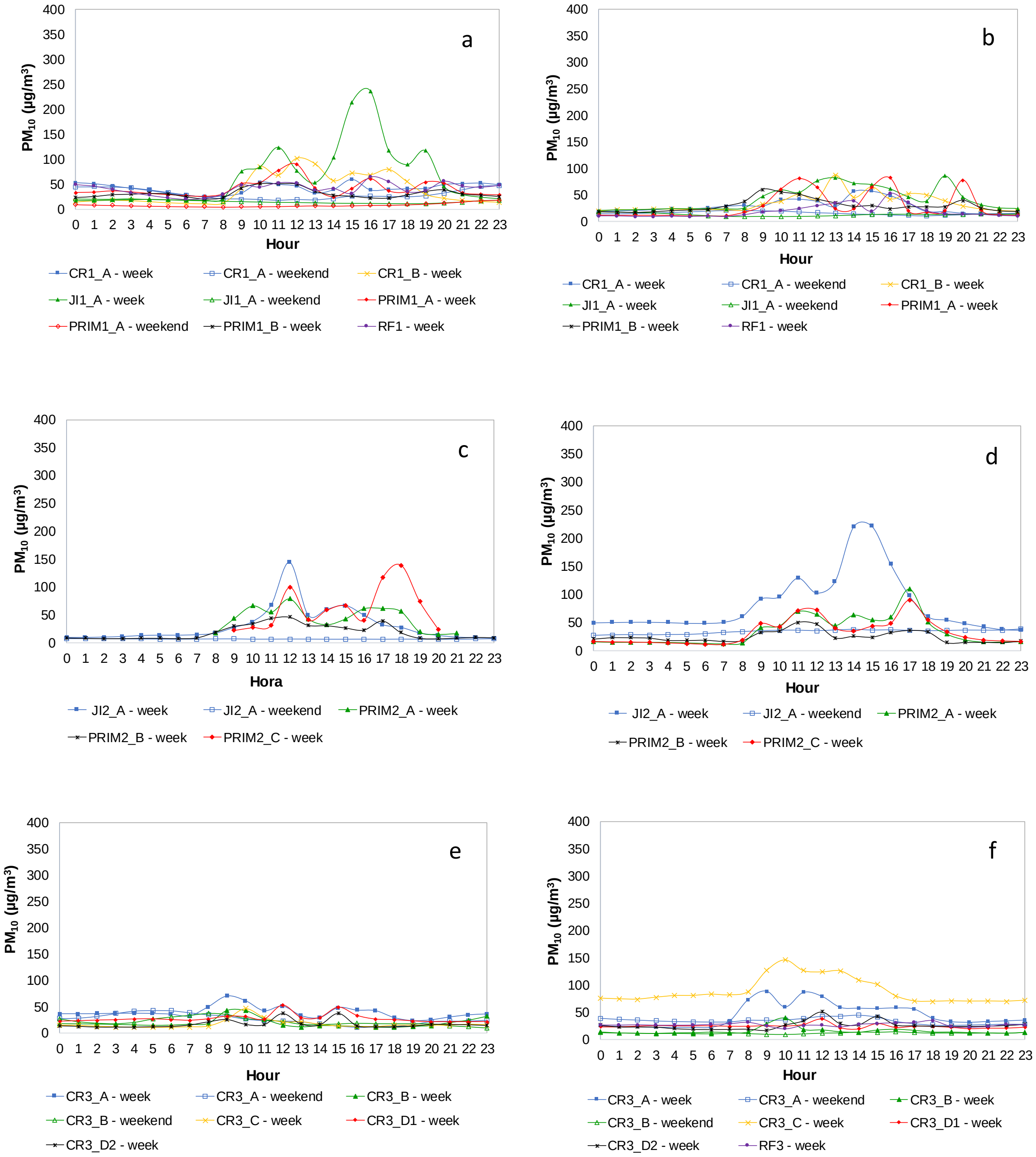

Figure S5. PM_{10} mean daily profile for: (**a**) Building 1—first campaign; (**b**) Building 1—second campaign (after IAP mitigation measures implemented); (**c**) Building 2—first campaign, (**d**) Building 2—second campaign (after IAP mitigation measures implemented); (**e**) Building 3—first campaign, (**f**) Building 3—second campaign (after IAP mitigation measures implemented).

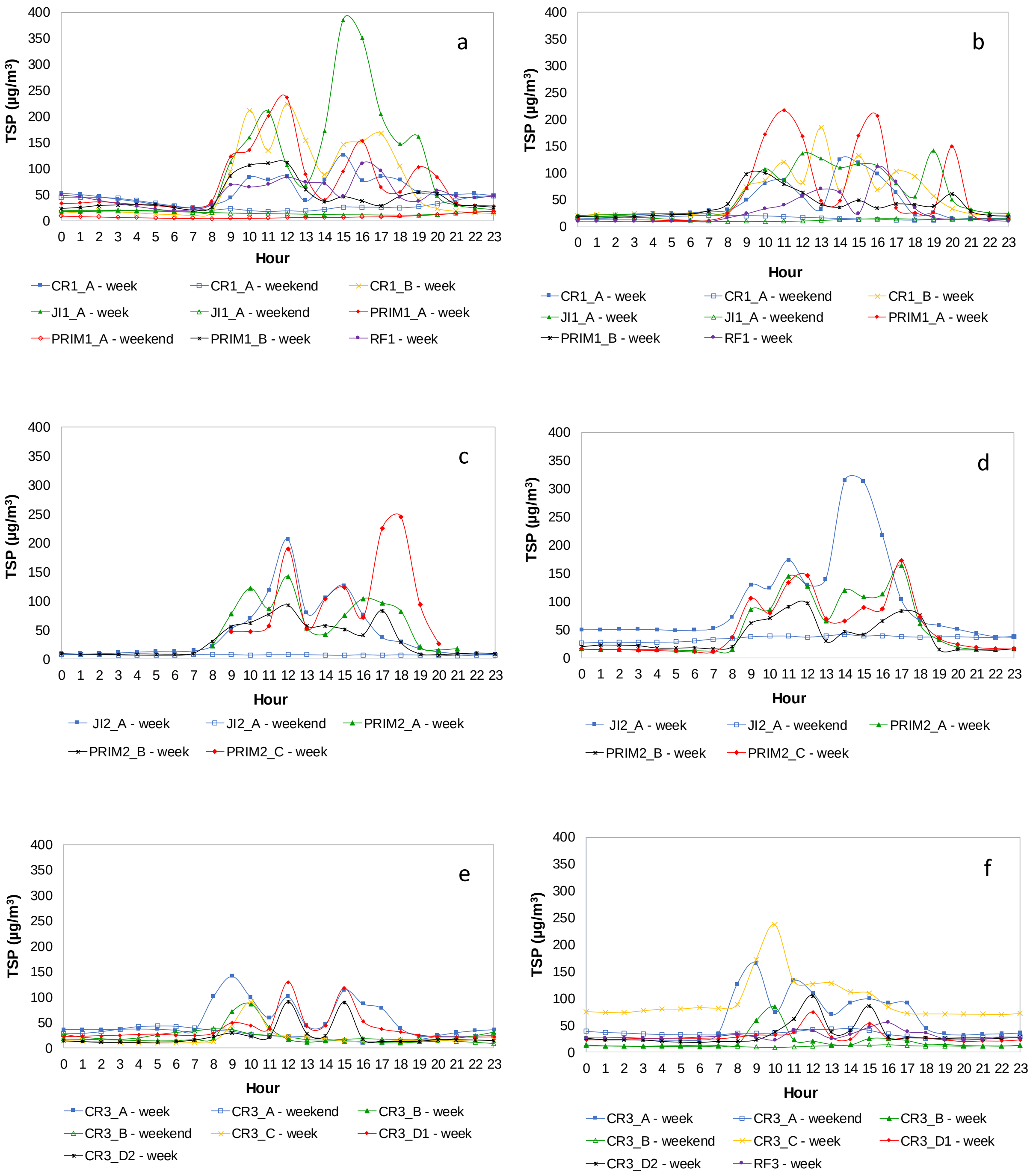

Figure S6. TSP mean daily profile for: (**a**) Building 1—first campaign; (**b**) Building 1—second campaign (after IAP mitigation measures implemented); (**c**) Building 2—first campaign, (**d**) Building 2—second campaign (after IAP mitigation measures implemented); (**e**) Building 3—first campaign, (**f**) Building 3—second campaign (after IAP mitigation measures implemented).

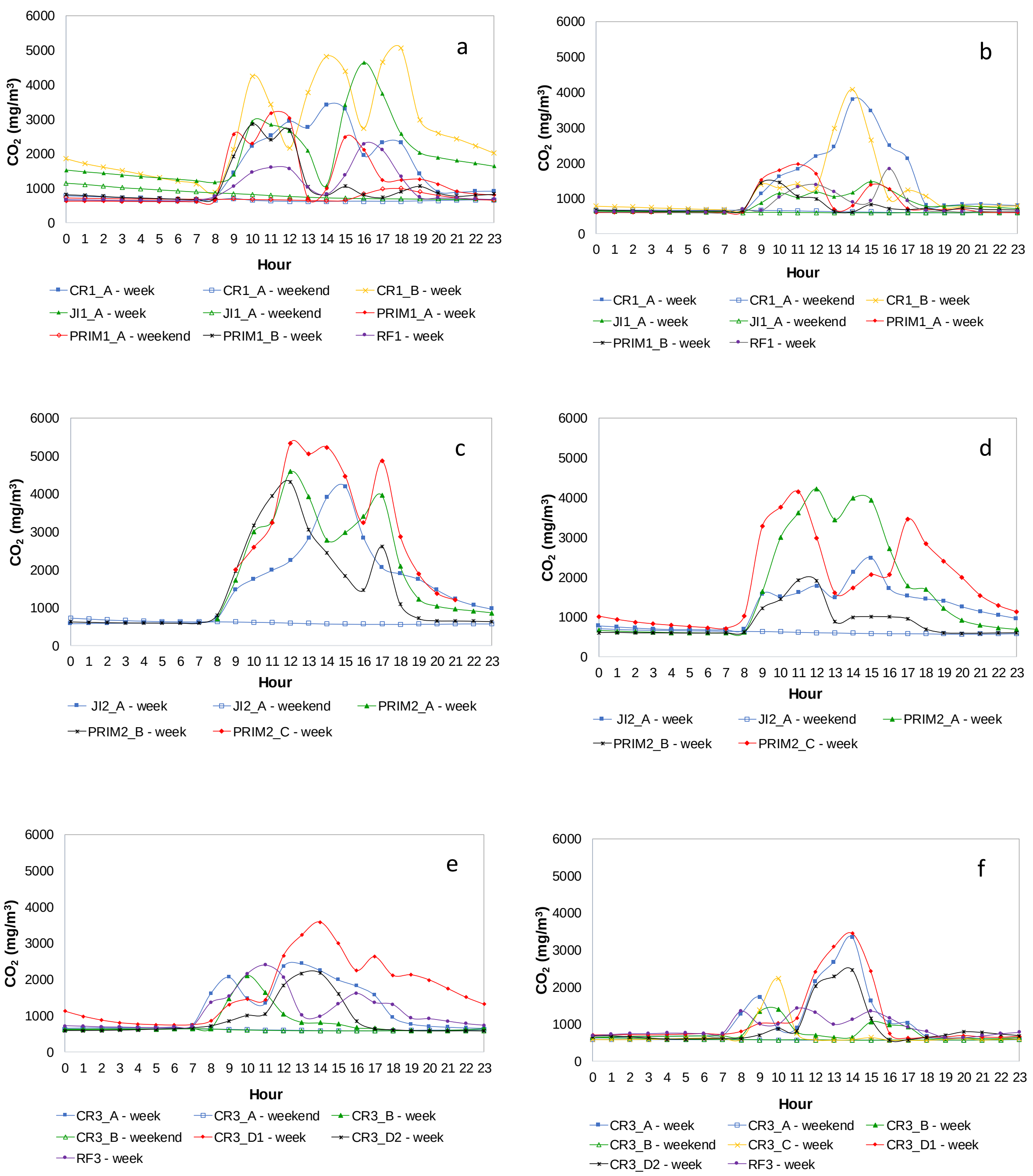

Figure S7. CO_2 mean daily profile for: (**a**) Building 1—first campaign; (**b**) Building 1—second campaign (after IAP mitigation measures implemented); (**c**) Building 2—first campaign, (**d**) Building 2—second campaign (after IAP mitigation measures implemented); (**e**) Building 3—first campaign, (**f**) Building 3—second campaign (after IAP mitigation measures implemented).

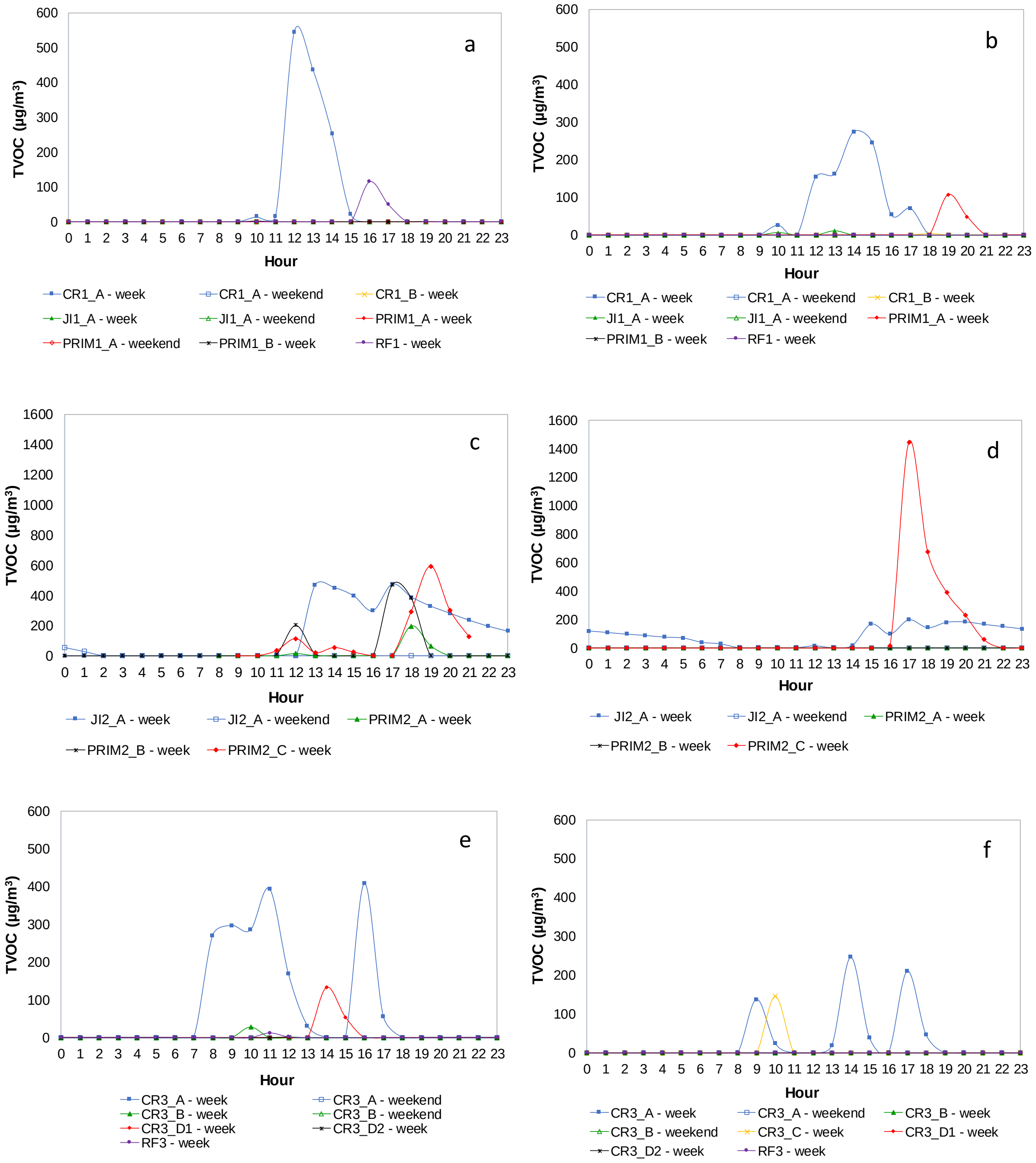

Figure S8. Total organic volatile compounds mean daily profile for: (**a**) Building 1—first campaign; (**b**) Building 1—second campaign (after IAP mitigation measures implemented); (**c**) Building 2—first campaign; (**d**) Building 2—second campaign (after IAP mitigation measures implemented); (**e**) Building 3—first c campaign, (**f**) Building 3—second campaign (after IAP mitigation measures implemented).

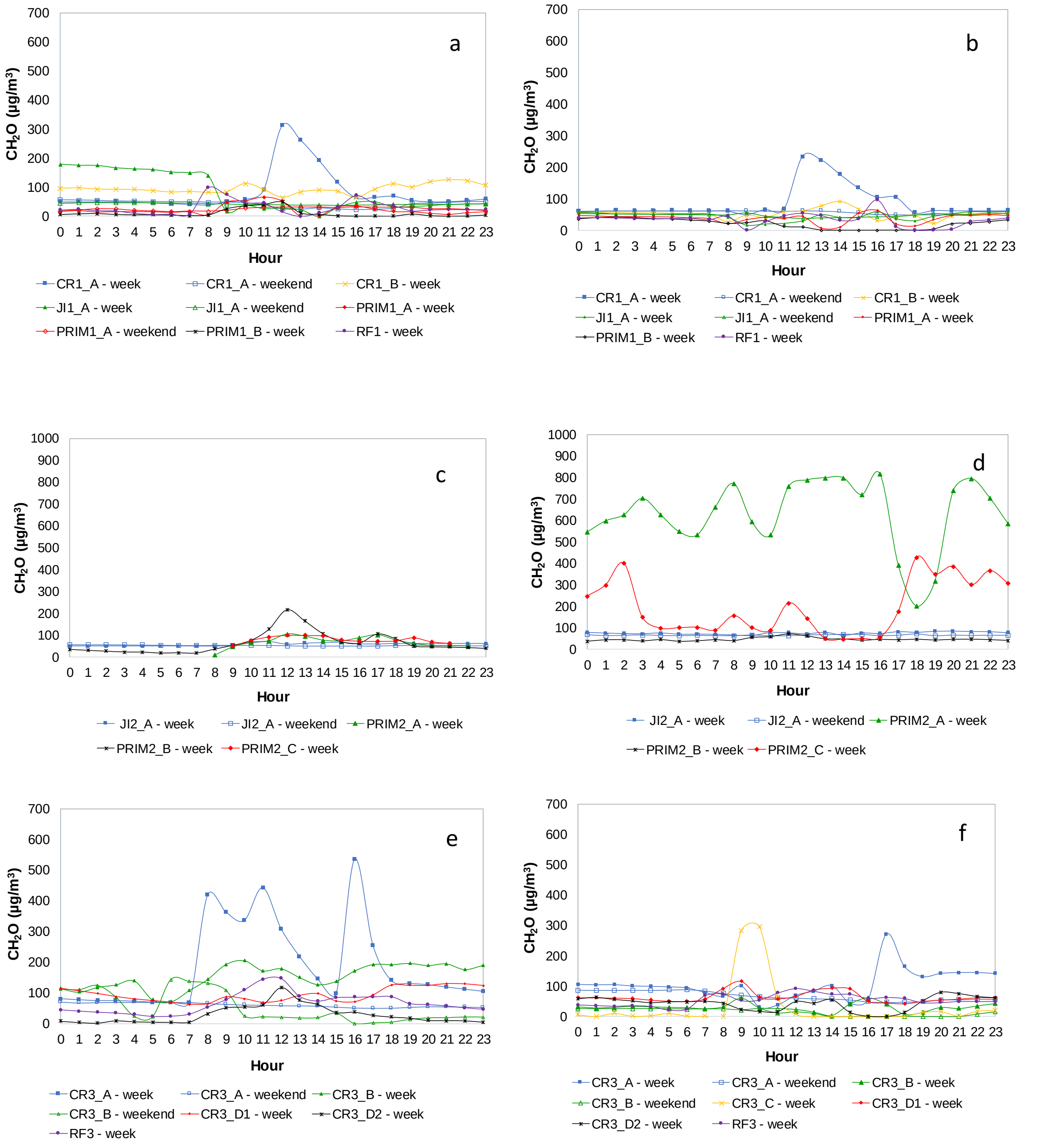

Figure S9. Formaldehyde mean daily profile for: (**a**) Building 1—first campaign; (**b**) Building 1—second campaign (after IAP mitigation measures implemented); (**c**) Building 2—first campaign; (**d**) Building 2—second campaign (after IAP mitigation measures implemented); (**e**) Building 3—first campaign, (**f**) Building 3—second campaign (after IAP mitigation measures implemented).

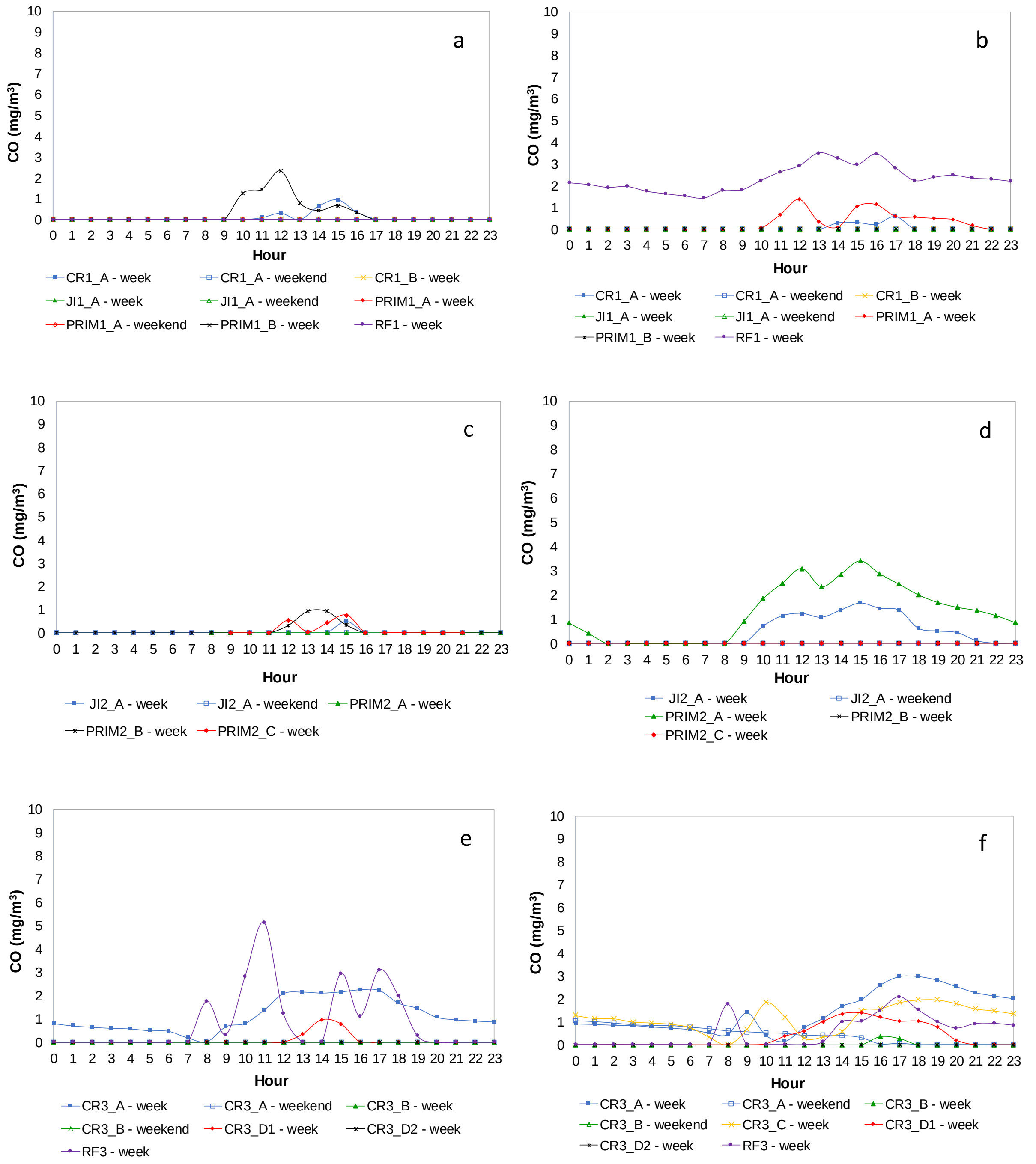

Figure S10. Carbone monoxide mean daily profile for: (**a**) Building 1—first campaign; (**b**) Building 1—second campaign (after IAP mitigation measures implemented); (**c**) Building 2—first campaign; (**d**) Building 2—second campaign (after IAP mitigation measures implemented); (**e**) Building 3—first campaign, (**f**) Building 3—second campaign (after IAP mitigation measures implemented).

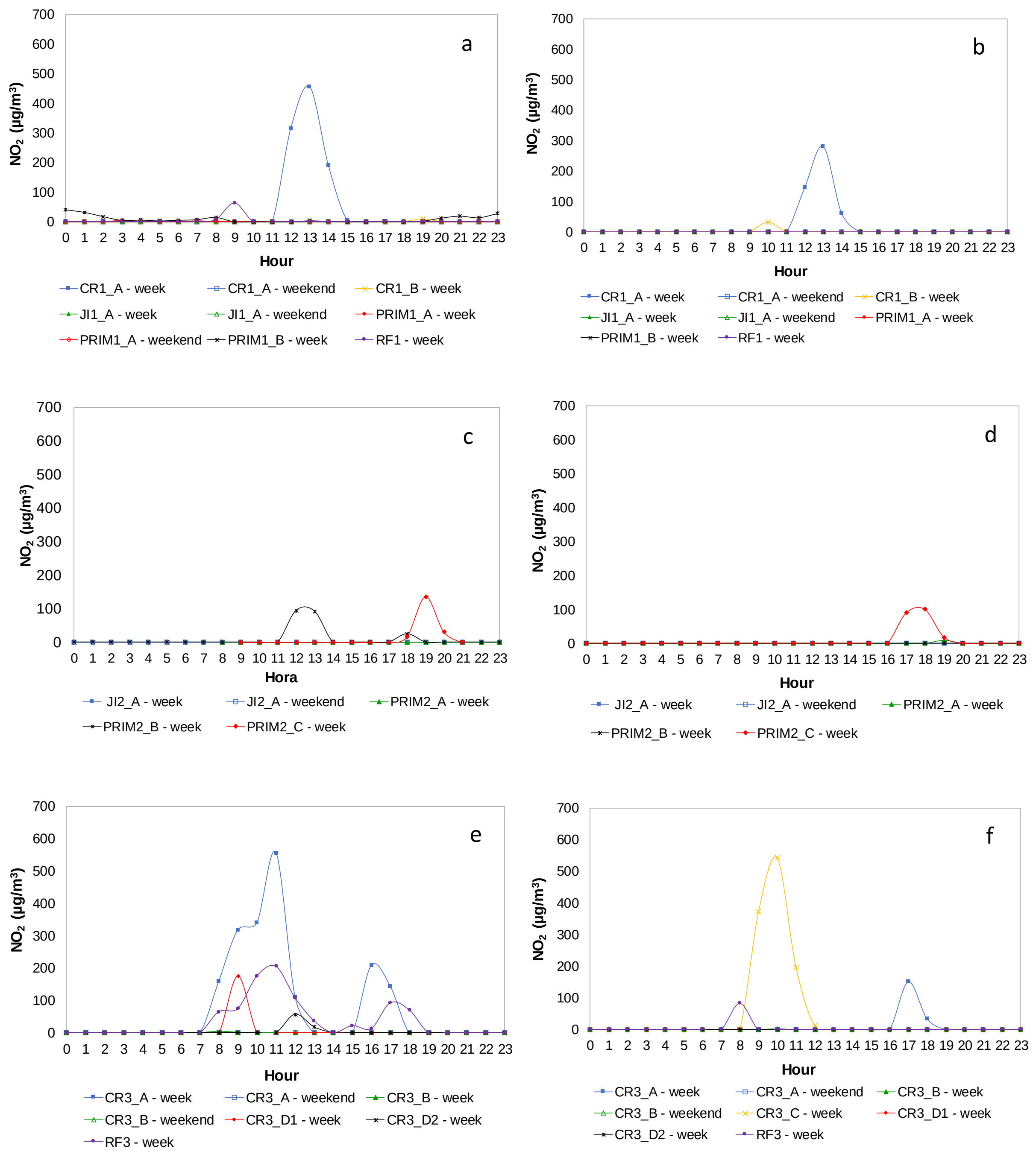

Figure S11. Nitrogen dioxide mean daily profile for: (**a**) Building 1—first campaign; (**b**) Building 1—second campaign (after IAP mitigation measures implemented); (**c**) Building 2—first campaign; (**d**) Building 2—second campaign (after IAP mitigation measures implemented); (**e**) Building 3—first campaign, (**f**) Building 3—second campaign (after IAP mitigation measures implemented).

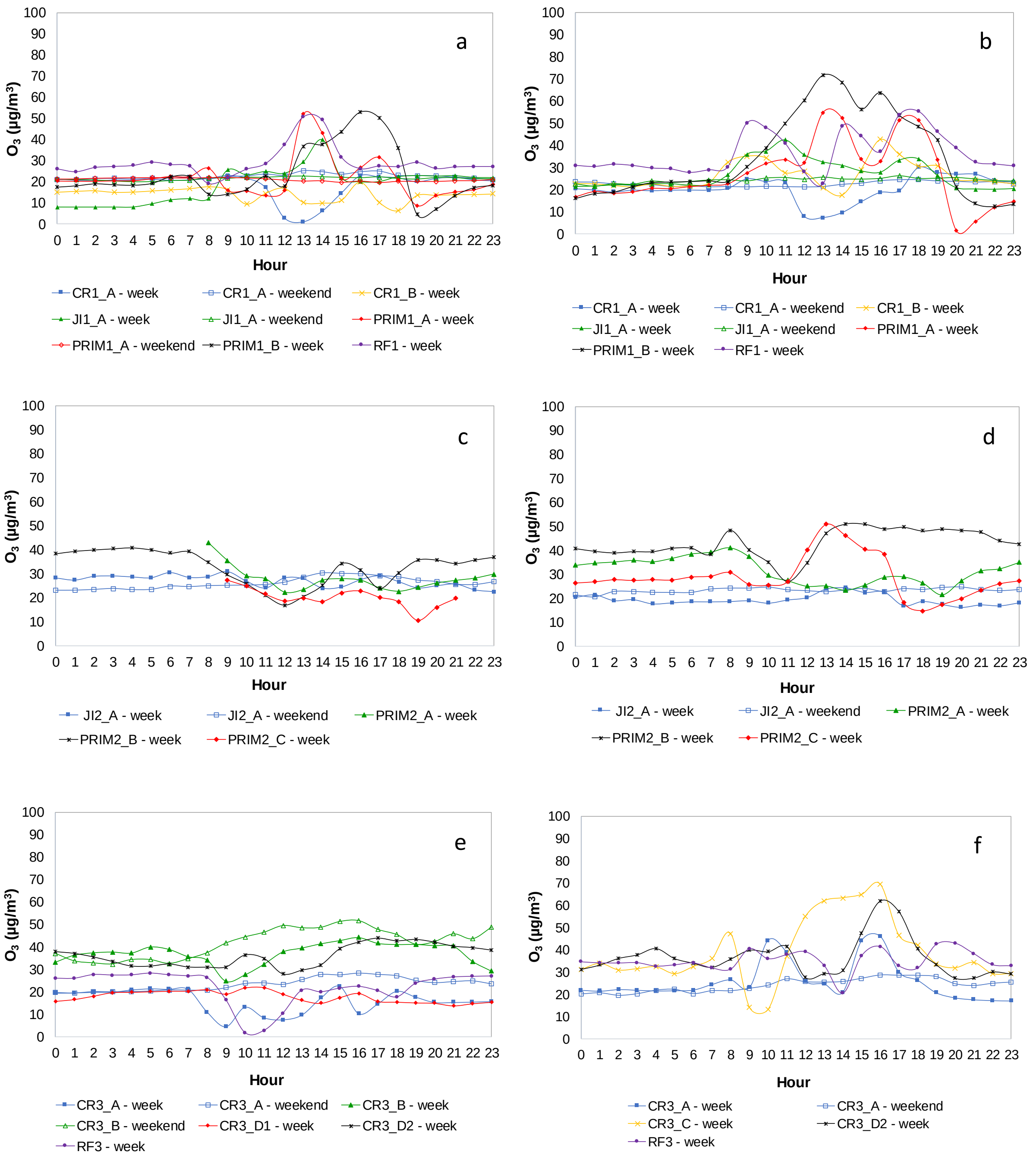

Figure S12. Ozone mean daily profile for: (**a**) Building 1—first campaign; (**b**) Building 1—second campaign (after IAP mitigation measures implemented); (**c**) Building 2—first campaign; (**d**) Building 2—second campaign (after IAP mitigation measures implemented); (**e**) Building 3—first campaign, (**f**) Building 3—second campaign (after IAP mitigation measures implemented).

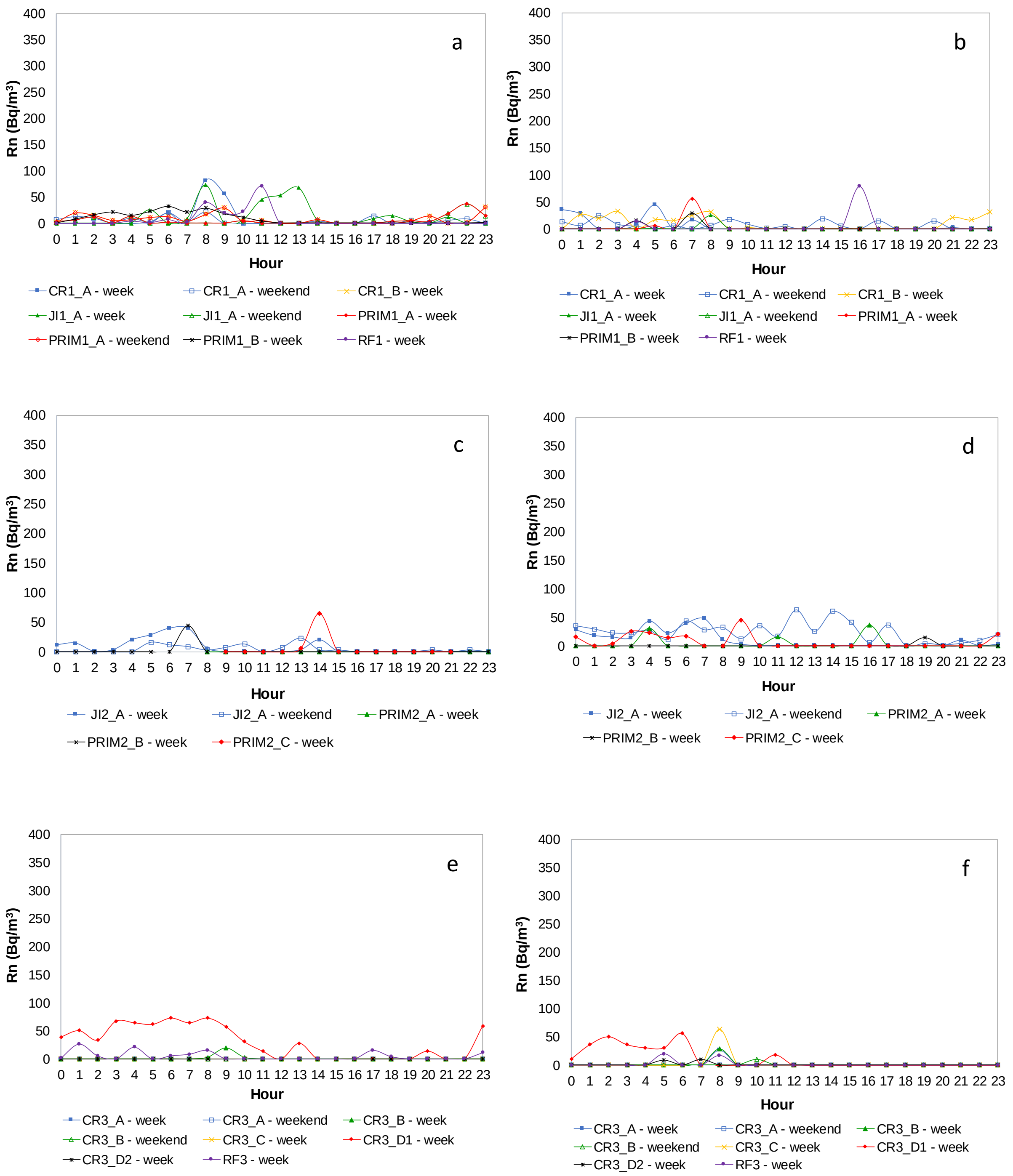

Figure S13. Radon mean daily profile for: (**a**) Building 1—first campaign; (**b**) Building 1—second campaign (after IAP mitigation measures implemented); (**c**) Building 2—first campaign; (**d**) Building 2—second campaign (after IAP mitigation measures implemented); (**e**) Building 3—first campaign, (**f**) Building 3—second campaign (after IAP mitigation measures implemented).
